# Designing a Multi‐Epitope Vaccine Against NOTCH1 and NOTCH4: A Computational Approach for Triple‐Negative Breast Cancer

**DOI:** 10.1155/bmri/9723304

**Published:** 2026-01-18

**Authors:** Pooriya Teimoori, Kosar Khatir, Mohammadreza Heidari

**Affiliations:** ^1^ Department of Biotechnology, School of Pharmacy, Alborz University of Medical Sciences, Karaj, Iran, abzums.ac.ir; ^2^ Department of Pharmaceutical Biotechnology, School of Pharmacy and Pharmaceutical Sciences, Isfahan University of Medical Sciences, Isfahan, Iran, mui.ac.ir; ^3^ Department of Clinical Pharmacy, School of Pharmacy, Alborz University of Medical Sciences, Karaj, Iran, abzums.ac.ir

**Keywords:** immunotherapy, NOTCH1, NOTCH4, triple-negative breast cancer, vaccine

## Abstract

Triple‐negative breast cancer (TNBC) has an aggressive nature, a specific set of molecular characteristics, distinct patterns of metastasis, and a lack of targeted treatment. Many types of cancer have Notch pathway dysregulation, which leads to tumor initiation, spreading, and increased therapeutic resistance. In breast cancer, the overexpression of NOTCH1 and NOTCH4 in tumors suggests their role as oncogenes. The Notch signaling pathway is highly active in breast cancer tissues and thus can be considered a possible target. This project is aimed at developing a protein‐based vaccine that targets NOTCH1 and NOTCH4 antigens associated with TNBC, using bioinformatic and in silico tools for increased precision, immunogenic potency, and faster therapeutic intervention development. The designed vaccine demonstrated coverage for 99.27%, indicating its potential effectiveness across diverse populations. Epitope‐MHC docking simulations demonstrated strong binding affinities, with docking scores ranging from −135.96 to −285.59, suggesting effective immune system activation. The immune modeling analysis suggested that the vaccine can induce a consistent and accurate immune response alongside an increase in immunoglobulins, B cells, memory T cells, and cytotoxic T cells. Physicochemical evaluations confirmed the vaccine′s stability, with an instability index of 39.70, indicating its robustness under physiological conditions. Furthermore, structural modeling of the vaccine indicated high stability and reliability under physiological conditions. Molecular docking demonstrated strong binding affinities with MHC I, MHC II, TLR4, and TLR7 molecules, with the highest docking score of −317.05 for TLR7 and the most favorable *Δ*G of −15.5 kcal/mol for TLR4. Molecular dynamics simulations (repeated three times) showed that the vaccine and its complexes with MHC I, MHC II, and TLR4 are stable, with the docked complexes exhibiting dynamic interaction. These findings collectively highlight a targeted approach to combating TNBC, demonstrating the vaccine′s potential as a therapeutic candidate.

## 1. Introduction

Breast cancer is the most prevalent kind of cancer, accounting for one in every eight reported newly diagnosed cases each year. Annually, there are 2.3 million new breast cancer diagnoses and 685,000 related deaths. The number of new cases is projected to rise by 40% and mortality by 50% over the next 2 decades, resulting in more than 3 million cases and 1 million deaths annually in 2040 [1]. Triple‐negative breast cancer (TNBC) is a distinct type of breast cancer characterized by the absence of progesterone receptor, human epidermal growth factor Receptor 2, and estrogen receptor. TNBC has an aggressive nature, a specific set of molecular characteristics, distinct patterns of metastasis, and a lack of targeted treatment. It accounts for around 10%–20% of invasive breast cancer instances, thereby prompting significant efforts to elevate its proportion of the overall breast cancer burden. Therefore, we should investigate potential treatments for TNBC, including targeted immunotherapy [2].

The Notch signaling pathway is highly conserved in communication among cells, regulating cell differentiation, proliferation, and apoptosis [3]. Many types of cancers have Notch pathway dysregulation, which leads to tumor initiation, spreading, and increased therapeutic resistance [4]. Therefore, it suggests an intriguing possibility for creating novel therapeutic methods since the Notch signaling system has been demonstrated to play essential roles in tumor formation and resistance [5]. The Notch family consists of four transmembrane receptors known as NOTCH1–4 [6]. The involvement of individual members of the Notch family in cancer can vary based on the specific cellular environment and kind of tumor. Increased NOTCH1 and NOTCH4 expressions have been observed in breast cancer. NOTCH1 and NOTCH4 have been recognized as oncogenes in many cancers [7]. NOTCH1 is important in breast cancer because it aids cancer cell growth and survival. NOTCH4 has a role in breast cancer through its involvement in angiogenesis and vascularization, which may influence tumor progression and metastasis [8].

Cancer immunotherapy may be divided into two categories: passive and active. Passive immunotherapy involves the use of immune components, including monoclonal antibodies such as trastuzumab and pertuzumab, which selectively target HER2 to inhibit cancer cell growth and replication. On the other hand, active immunotherapy wants to boost a more robust immune response from the patients so they can more successfully recognize and kill cancer. Cancer vaccines are authorized for specific malignancies caused by hepatitis B and human papillomavirus viruses, and they detect and eradicate cancer cells [9].

Tumor antigens such as HER2, p53, MUC1, CEA, and hTERT are expressed in various cancers, making them promising candidates for vaccine development [10]. Multiple platforms exist for in silico cancer vaccine development, encompassing DNA, RNA, dendritic cell‐based, and epitope ensemble vaccines. These platforms utilize computational prediction and experimental validation to identify and target tumor‐associated antigens, potentially revolutionizing cancer immunotherapy by providing safe and effective treatments [11].

This project uses in silico methods to design a protein‐based vaccine targeting TNBC‐associated NOTCH1 and NOTCH4 antigens. The objective is to enhance precision and immunogenicity while accelerating the development of a potentially effective therapeutic intervention.

## 2. Material and Methods

### 2.1. Sequence Retrieval

The amino acid sequences of the NOTCH1 and NOTCH4 proteins were acquired from the UniProt server (https://www.uniprot.org/; accessed on January 8, 2024) [12] (NOTCH1 ID: P46531 and NOTCH4 ID: Q99466). Antigenicity was assessed using the VaxiJen 2.0 server (http://www.ddg-pharmfac.net/vaxijen/VaxiJen/VaxiJen.html; accessed on January 8, 2024) [13–15], and membrane protein characteristics were evaluated using the TMHMM‐2.0 server (https://services.healthtech.dtu.dk/services/TMHMM-2.0/; accessed on January 8, 2024) [16, 17]. The three‐dimensional structure of proteins was acquired from the Swiss Model server (https://swissmodel.expasy.org/; accessed on January 8, 2024) [18–22] for NOTCH4 and the AlphaFold server (https://alphafold.ebi.ac.uk/; accessed on January 8, 2024) [23] for NOTCH1.

### 2.2. Epitope Prediction and Optimal Selection

#### 2.2.1. Prediction of T Cell Epitopes

The Immune Epitope Database (IEDB) (https://www.iedb.org/; accessed on January 8, 2024) [24] is distinguished in predicting MHC I and II epitopes by employing validated methods that ensure accurate binding prediction for both MHC classes. Its adaptable tools facilitate the discovery of new B and T cell epitopes, incorporating molecular target prediction and analysis. The IEDB consensus server utilizes a percentile rank strategy, suggesting selecting the Top 20% of predictions, encompassing 50% of the immune response. This approach integrates multiple machine learning prediction algorithms to improve the precision of MHC I and II epitope predictions [25, 26].

##### 2.2.1.1. MHC I (CTL) Epitope Prediction

IEDB NetMHCpan EL 4.1 (https://services.healthtech.dtu.dk/services/NetMHCpan-4.1/; accessed on January 8, 2024) [27] and Consensus servers [28] were employed with a percentile rank below two to predict MHC I epitopes. The NetMHCPan server utilizes artificial neural networks (ANNs) trained on sequences of MHC molecules for epitope prediction [29]. Epitopes were predicted for several HLAs, including HLA‐A∗01:01, HLA‐A∗02:01, HLA‐A∗02:06, HLA‐A∗03:01, HLA‐A∗24:02, HLA‐A∗26:01, HLA‐B∗07:02, HLA‐B∗08:01, HLA‐B∗27:05, HLA‐B∗39:01, HLA‐B∗44:02, HLA‐B∗44:03, HLA‐B∗58:01, and HLA‐B∗58:02. The length of epitopes was set to nine. Then, the epitopes were screened by VaxiJen 2.0 (the threshold limit was 0.5), AllerCatPro 2.0 (https://allercatpro.bii.a-star.edu.sg/; accessed on January 8, 2024) [30], and ToxinPred (https://webs.iiitd.edu.in/raghava/toxinpred/algo.php; accessed on January 8, 2024) [31, 32] servers in terms of antigenicity, allergenicity, and toxicity, respectively. In the context of the ToxinPred server, the SVM (Support Vector Machine) method was employed with a foundation in Swiss‐Prot data. The SVM threshold was established at 0. In addition, certain physicochemical properties were shown for a complete protein characterization. These included hydropathicity, hydrophobicity, hydrophilicity, charge, and molecular weight. The IEDB Class I immunogenicity tool (http://tools.iedb.org/immunogenicity/; accessed on January 8, 2024) [33] was also employed to assess epitope immunogenicity, utilizing a masking strategy that involves default positions (first, second, and C‐terminus amino acids) and applying a threshold criterion with a requirement for a score greater than 0.

##### 2.2.1.2. MHC II (HTL) Epitope Prediction

IEDB Consensus [34] (percentile rank < 2) and NetMHCIIpan‐4.0 servers (https://services.healthtech.dtu.dk/services/NetMHCIIpan-4.0/; accessed on January 8, 2024) [35] (A threshold of 1% was set for defining strong binding and 5% for weak binding interactions) were used to predict MHC II epitopes. Epitope prediction was conducted using the complete DR, DP, and DQ alleles (the threshold limit was 0.5). Predicted epitopes were screened using VaxiJen 2.0, AllerCatPro 2.0, and ToxinPred servers for antigenicity, allergenicity, and toxicity, respectively. Subsequently, IFNepitope (http://crdd.osdd.net/raghava/ifnepitope/; accessed on January 8, 2024) [36] and IL4pred (http://crdd.osdd.net/raghava/il4pred/; accessed on January 8, 2024) [37] servers were utilized to identify epitopes that induced interferon‐gamma (INF‐G) and interleukin‐4 responses. For the IFNepitope server, the approach for predicting IFN‐gamma epitopes was (Motif and SVM hybrid), and the prediction model was (IFN‐gamma versus non‐IFN‐gamma/IFN‐gamma versus another cytokine). For the IL4pred server, the prediction model chosen for epitope analysis was a hybrid approach combining SVM and motif methods. An SVM threshold of 0.2 was set, and the displayed physicochemical properties included hydrophobicity, hydropathicity, hydrophilicity, charge, and molecular weight. After that, MHC I epitopes coexisting within MHC II epitope sequences were eliminated, leaving only MHC II epitopes that included MHC I epitopes. Finally, four epitopes with the highest MHCI and MHC II antigenicity were selected.

#### 2.2.2. LBL Epitope Prediction

B cell linear epitopes were predicted using the IEDB antigen sequence properties (http://tools.iedb.org/bcell/; accessed on January 8, 2024) and ABCpred server (https://webs.iiitd.edu.in/raghava/abcpred/ABC_submission.html; accessed on January 8, 2024) [38, 39]. The ABCpred server employs ANNs to predict B cell epitopes within antigens, contributing to identifying potential vaccine candidates, disease diagnosis, and allergy research. Notably, its accuracy has been reported as 67.9% [40]. A threshold value of 0.50 was defined, and the length of epitopes was set to 16 amino acids. Within the IEDB server, a threshold of 0.7 was applied alongside utilizing the “Bepipred Linear Epitope Prediction 2.0” method [41–43]. Like T cell epitope prediction, VaxiJen 2.0, AllerCatPro 2.0, and ToxinPred servers (with the same criteria as before) were employed to assess B cell epitopes. Next, epitopes were classified based on their antigenicity, and the Top 4 epitopes with the highest antigenicity were chosen.

### 2.3. Population Coverage Analysis

Considering the diversity of MHC structures among populations, evaluating the coverage of the identified epitopes across populations is crucial. To accomplish this, the IEDB Population Coverage tool was employed (http://tools.iedb.org/population/; accessed on January 8, 2024) [44].

### 2.4. Epitope Docking

An epitope docking test with MHCs was carried out. HDOCK server (http://hdock.phys.hust.edu.cn/; accessed on November 2, 2024) [45–50] was utilized to dock MHC I epitopes with MHC A 02 (PDB ID: 3MRG) and A 03 (PDB ID: 2XPG) proteins and MHC II epitopes with MHC DRB1 0101 (PDB ID: 1AQD) protein. HDOCK employs a hybrid algorithm that combines template‐based modeling and ab initio free docking to predict interactions between proteins and proteins or proteins and DNA [51].

### 2.5. Vaccine Construction

EAAAK and GPGPG linkers were used as linkers of selected epitopes in the vaccine construct. A KK linker was also used in the structure. We added granulocyte‐macrophage colony‐stimulating factor (GM‐CSF) as an adjuvant to the vaccine design.

### 2.6. Analysis of Physicochemical Properties and Posttranslational Modifications

#### 2.6.1. Assessment of Physicochemical Characteristics

The properties of the developed vaccine were assessed using the ProtParam server (https://web.expasy.org/protparam/; accessed on January 8, 2024). ProtParam provides efficient methods for analyzing protein physicochemical properties. It includes calculations for molecular weight, representing a protein′s atomic weight sum; isoelectric point, indicating pH neutrality; amino acid composition, providing amino acid frequency; and extinction coefficient, estimating protein concentration from UV absorbance. These methods support protein characterization and experimentation [52].

#### 2.6.2. Evaluation of Posttranslational Alterations

Phosphorylation, acetylation, and N‐glycosylation analyses were conducted using the NetPhos‐3.1 (https://services.healthtech.dtu.dk/services/NetPhos-3.1/; accessed on January 8, 2024) [53], NetAcet‐1.0 (https://services.healthtech.dtu.dk/services/NetAcet-1.0/; accessed on January 8, 2024) [54], and NetNGlyc‐1.0 (https://services.healthtech.dtu.dk/services/NetNGlyc-1.0/; accessed on January 8, 2024) [55] servers, respectively. The Big‐PI/GPI Animals server (https://mendel.imp.ac.at/gpi/gpi_server.html; accessed on January 8, 2024) [56–58] was employed to predict the glycosylphosphatidylinositol (GPI) modification site. Further, the MyrPS/NMT server (https://mendel.imp.ac.at/myristate/; accessed on January 8, 2024) [59] was used to predict the N‐terminal N‐Myristoylation of the constructed vaccine.

### 2.7. Prediction of Secondary Structure

Accurate secondary structure prediction is crucial in vaccine design, enabling precise epitope mapping and stability assessments to create effective and stable candidates. Using PSIPRED (http://bioinf.cs.ucl.ac.uk/psipred/; accessed on November 2, 2024), the vaccine model′s structure was predicted with a two‐stage neural network, achieving a Q3 accuracy of 76.5% to 78.3% through PSI‐BLAST matrices [60].

### 2.8. 3D Modeling and Validation of Vaccine

#### 2.8.1. 3D Model Prediction

3D protein structure prediction was done by the Robetta server (https://robetta.bakerlab.org/submit.php; accessed on January 8, 2024) [61]. Robetta is an automated server for predicting protein structures, employing the Rosetta fragment insertion method. It integrates template‐based and ab initio approaches for accurate protein structure predictions [62]. Following this, the GalaxyWEB server (https://galaxy.seoklab.org/cgi-bin/submit.cgi?type=REFINE; accessed on November 2, 2024) [63, 64] was used to refine the model. GalaxyRefine utilizes molecular dynamics (MD) simulation to conduct iterative changes in the structure, followed by an overall structural relaxation. This process explicitly targets clusters of side chains for structure perturbation.

#### 2.8.2. Analysis of Vaccine

The assessment of the constructed 3D model was conducted using the SAVES servers (https://saves.mbi.ucla.edu/; accessed on November 2, 2024), which consist of ERRAT [65], ProCheck [66], Verify3D [67] tools, and also the ProSa‐web server (https://prosa.services.came.sbg.ac.at/prosa.php; accessed on November 2, 2024) [68, 69]. ERRAT is a protein structure validation tool that assesses atom–atom interactions, measuring structural quality. It employs statistical analysis and sliding window calculations [65]. Verify3D assesses protein models by classifying residues into structural environments, helping detect global errors. It relies on three‐dimensional profiles to match sequences, indicating model quality [67]. ProCheck assesses protein stereochemical quality through geometry checks. Its algorithms evaluate overall and residue‐specific properties. It also utilizes Ramachandran plots to depict the validity of dihedral angles in protein structures [66]. ProSA‐web employs mean force potentials to assess protein structures, using z‐scores and energy plots to detect errors and validate deviations from native protein structures based on an extensive database of known protein structures [69]. In addition, the antigenicity and allergenicity of the substances were assessed using the VaxiJen 2.0 and AllerCatPro 2.0 servers, respectively.

### 2.9. Prediction of Discontinuous B Cell Epitopes

B cell discontinuous epitopes were predicted using the IEDB ElliPro server (http://tools.iedb.org/ellipro/; accessed on January 8, 2024) [70]. ElliPro integrates ellipsoid approximation, residue protrusion index calculation, and PI‐based clustering algorithms to represent protein structures and predict antibody epitopes. Its 82% ± 0.05 accuracy suggests high precision, validating its efficacy in epitope identification within the dataset [70].

### 2.10. Immune Simulation Analysis

The C‐ImmSim server (https://kraken.iac.rm.cnr.it/C-IMMSIM/; accessed on January 8, 2024) was utilized to predict how the vaccine construct will stimulate the immune system following injection. The C‐ImmSim server employs the Celada–Seiden model for dynamic immune simulation, predicting immune stimulation responses to proteins, aiding vaccine design, and therapeutic strategies [71]. The C‐ImmSim server was employed with specific parameter settings: a random seed of 12345, a simulation volume of 10, simulation steps set to 540, and host HLA selection including A0101 and B0702 for MHC Class I and DRB1: 0101 for MHC Class II. The vaccine was administered in three doses, with a 28‐day interval between injections.

### 2.11. Molecular Docking

Molecular docking studies confirmed the interaction between the constructed vaccine and its receptors. The constructed vaccine was docked against various molecules, namely, TLR4 (PDB ID: 4G8A), TLR7 (PDB ID: 7CYN), HLA A 0201 (PDB ID: 3MRG), HLA A 0301 (PDB ID: 2XPG), and HLA DRB1 0101 (PDB ID: 1AQD) through the HDOCK server. Following this, the Prodigy server (https://wenmr.science.uu.nl/prodigy/; accessed on January 8, 2024) [72, 73] was utilized to compute Gibbs free energy (*Δ*G) and dissociation constant (Kd). The Prodigy server utilizes a contact‐based method to predict protein–protein complex binding affinity, considering intermolecular contacts and percentages of charged and polar amino acids. It also estimates the change in *Δ*G for the protein–protein interactions. Finally, PDBsum (https://www.ebi.ac.uk/thornton-srv/databases/pdbsum/Generate.html; accessed on November 2, 2024) provides structural summaries and detailed interaction analyses of docked protein structures, including hydrogen bonds (H‐bonds), nonbonded contacts, and salt bridges, aiding in understanding complex molecular interactions. This server was useful for assessing vaccine structures docked with MHC I, MHC II, TLR4, and TLR7, offering insights into binding mechanisms and structural quality [74].

### 2.12. MD

MD analysis gained relevance for vaccine design by elucidating specific insights into molecular interactions, structural stability, and dynamics within nonrigid molecular systems. The approach serves to refine candidates for a vaccine by predicting behavior under physiological conditions, thereby enhancing their immunogenicity and potential efficacy. In this study, GROMACS 2024.4 was employed alongside the CHARM36 force field for topology generation, providing a reliable framework for simulating molecular interactions. The SPC water model was used within triclinic box types, and a salt concentration of 0.15 M NaCl was maintained to mimic physiological conditions. The system underwent energy minimization, servicing multiple equilibrations under NVT and NPT ensembles at temperatures of 300 K and 1 bar pressure. The MD simulation progressed in a leapfrog manner over 50 ns and yielded roughly 5000 frames [75, 76]. The MD analysis was repeated three times to ensure the robustness of the results.

#### 2.12.1. Root Mean Square Deviation (RMSD)

RMSD is the quantity that provides an average measure of the deviation of the atomic coordinates between two molecular structures, which signals conformational stability with time. Low values indicate few deviations; hence, high structural stability.

#### 2.12.2. Root Mean Square Fluctuation (RMSF)

During MD simulations, RMSF measures the average distance between atomic positions and their average position. RMSF provides insight into the flexibility of certain atoms or regions of a molecule, thus illustrating areas of greater mobility or instability within the overall system.

#### 2.12.3. Radius of Gyration (Rg)

The Rg, which quantifies the roundness of a molecule, is defined as the square root of the mean of all the squares of the distances from the center of mass to the particle forming that molecule. In MD, Rg is employed to analyze the stability of a structure concerning time‐derived events occurring for temporally folded states of a given protein or complex.

#### 2.12.4. Solvent Accessible Surface Area (SASA)

SASA describes solvent‐accessible surface area. It provides information regarding any conformational changes that might happen within a molecule. The SASA gains insight into protein folding and dynamics and interaction with ligands or other biomolecules, which is key to evaluating molecular behavior in biomolecule solutions.

#### 2.12.5. H‐Bond

H‐bond operationally means that the number and stability of the H‐bonding between interacting molecules are assessed during MD simulation. The number and lifetime of H‐bonds will give information about the stability of the given molecule, the interaction of binding, and variations in conformation. The fluctuations in the number of H‐bonds can suggest dynamic processes such as ligand binding or protein folding.

## 3. Results

### 3.1. Retrieval and Analysis of Target Proteins

The protein sequences for NOTCH1 and NOTCH4 were retrieved from the UniProt server. VaxiJen 2.0 and TMHMM 2.0 analyses revealed that NOTCH1 and NOTCH4 are present on the cell surface and have antigenic properties (Table 1).

**Table 1:** Analysis of target proteins.

**Number**	**Protein name**	**Accession number**	**Amino acids**	**Antigenicity**	**Extracellular domain**
1	NOTCH1	P46531	2555	0.4479 (Antigen)	19–1735
2	NOTCH4	Q99466	2003	0.4191 (Antigen)	24–1447

### 3.2. Epitope Prediction and Assessment

#### 3.2.1. "MHC I (CTL)" Epitope Prediction

From the IEDB servers, 243 epitopes for NOTCH1 and 241 epitopes for NOTCH4 were acquired, all with a percentile rank lower than two. A total of 102 NOTCH1 epitopes and 111 NOTCH4 epitopes with antigenicity values greater than 0.5 passed the antigenicity test through the VaxiJen 2.0 server. Out of these, 92 epitopes of NOTCH1 and 58 epitopes of NOTCH4 were identified as nontoxic and nonallergenic by the ToxinPred and AllerCatPro 2.0 servers. After that, the immunogenicity of the epitopes was analyzed using the IEDB Class I immunogenicity tool, and it was determined that 45 epitopes of NOTCH1 and 27 epitopes of NOTCH4 were indeed valid. Finally, based on the highest antigenicity, four epitopes of NOTCH1 and NOTCH4 were chosen (Table 2).

**Table 2:** Selected CTL epitope analysis.

**CTL epitopes**						
**Protein**	**Number**	**Sequence**	**Antigenicity**	**Toxicity**	**Immunogenicity**	**Allergenicity**
NOTCH1	1	DQIGEFQCI	2.2717	−0.53	0.15574	Not allergen
	2	CDLLTLTEY	1.8650	−1.06	0.10386	Not allergen
	3	CEWDGLDCA	1.8617	−0.07	0.104	Not allergen
	4	CRAGHTGRR	1.7658	−0.70	0.17368	Not allergen

NOTCH4	5	CQLRDFCSA	1.6345	−0.70	0.03812	Not allergen
	6	DLLNGFQCI	1.4877	−0.78	0.00383	Not allergen
	7	CSLGVPDPW	1.1756	−0.73	0.0725	Not allergen
	8	GQEGPRCEL	1.1667	−0.65	0.11752	Not allergen

#### 3.2.2. MHC II (HTL) Epitope Prediction

Analysis of NOTCH1 and NOTCH4 proteins by IEDB and NetMHCIIpan‐4.0 servers to predict HTL epitopes generated 312 unique epitopes. Of these 312 epitopes, 149 obtained appropriate antigenicity in the analysis by the VaxiJen 2.0 server. In the next step, by evaluating the toxicity and allergenicity of epitopes using ToxinPred and AllerCatPro 2.0 servers, 95 epitopes were approved. The INF epitope server confirmed that 27 could induce INF‐gamma out of these epitopes. Out of the total 27 epitopes, the IL4Pred server verified that only 10 epitopes associated with NOTCH1 are identified as stimulants for IL4. At the same time, no confirmation was found for any of the epitopes related to NOTCH4. The final four epitopes of each protein were selected based on the highest antigenicity (Table 3).

**Table 3:** Selected HTL epitope analysis.

**HTL epitopes**							
**Protein**	**Number**	**Sequence**	**Antigenicity**	**Toxicity**	**Allergenicity**	**INF*γ***	**IL4**
NOTCH1	1	SSFHFLRELSRVLHT	1.0456	−0.57	Not allergen	0.20223948	0.28
	2	SFHFLRELSRVLHTN	1.0442	−0.74	Not allergen	0.14618787	0.28
	3	RNSSFHFLRELSRVL	1.0214	−0.59	Not allergen	0.015396429	0.29
	4	FHFLRELSRVLHTNV	1.0055	−0.75	Not allergen	0.297036	0.28

NOTCH4	5	ARAEEKLGGTRDPTY	1.3792	−0.84	Not allergen	0.17267817	−0.73
	6	PLLAVHPHAGTAPPA	0.6919	−1.01	Not allergen	0.02993192	−0.84
	7	TYQERAAPQTQPLGK	0.4922	−1.40	Not allergen	0.32824736	−1.22
	8	PWDPGLLLRFLAAMA	0.4744	−1.16	Not allergen	0.54342203	−0.74

#### 3.2.3. LBL Epitope Prediction

The prediction of B cell epitopes involved using two servers, namely, IEDB and ABCpred. This computational analysis yielded a total of 393 distinct epitopes. The VaxiJen 2.0 server analyzed these epitopes, of which 207 exhibited the desired level of antigenicity. The ToxinPred and AllerCatPro 2.0 servers evaluated epitopes, resulting in 43 out of the remaining 207 epitopes being identified as nontoxic and nonallergenic. The four epitopes with the greatest antigenicity were selected for each protein (Table 4).

**Table 4:** Selected LBL epitope analysis.

**LBL epitopes**					
**Protein**	**Number**	**Sequence**	**Antigenicity**	**Toxicity**	**Allergenicity**
NOTCH1	1	LPGGSEGGRRRRELDP	1.2650	−1.00	Not allergen
	2	CHILDYSFGGGAGRDI	1.1785	−1.13	Not allergen
	3	EQLRNSSFHFLRELSR	1.1705	−0.79	Not allergen
	4	EWDGLDCAEHVPERLA	0.9820	−‐0.96	Not allergen

NOTCH4	5	KLGGTRDPTYQERAAP	1.0246	−1.10	Not allergen
	6	GWTGEQCQLRDFCSAN	0.9369	−0.67	Not allergen
	7	GSTCIDRVGSFSCLCP	0.8678	−0.57	Not allergen
	8	TCLSLSLGQGTCQCAP	0.7249	−0.52	Not allergen

### 3.3. Population Coverage

**Figure 1:**
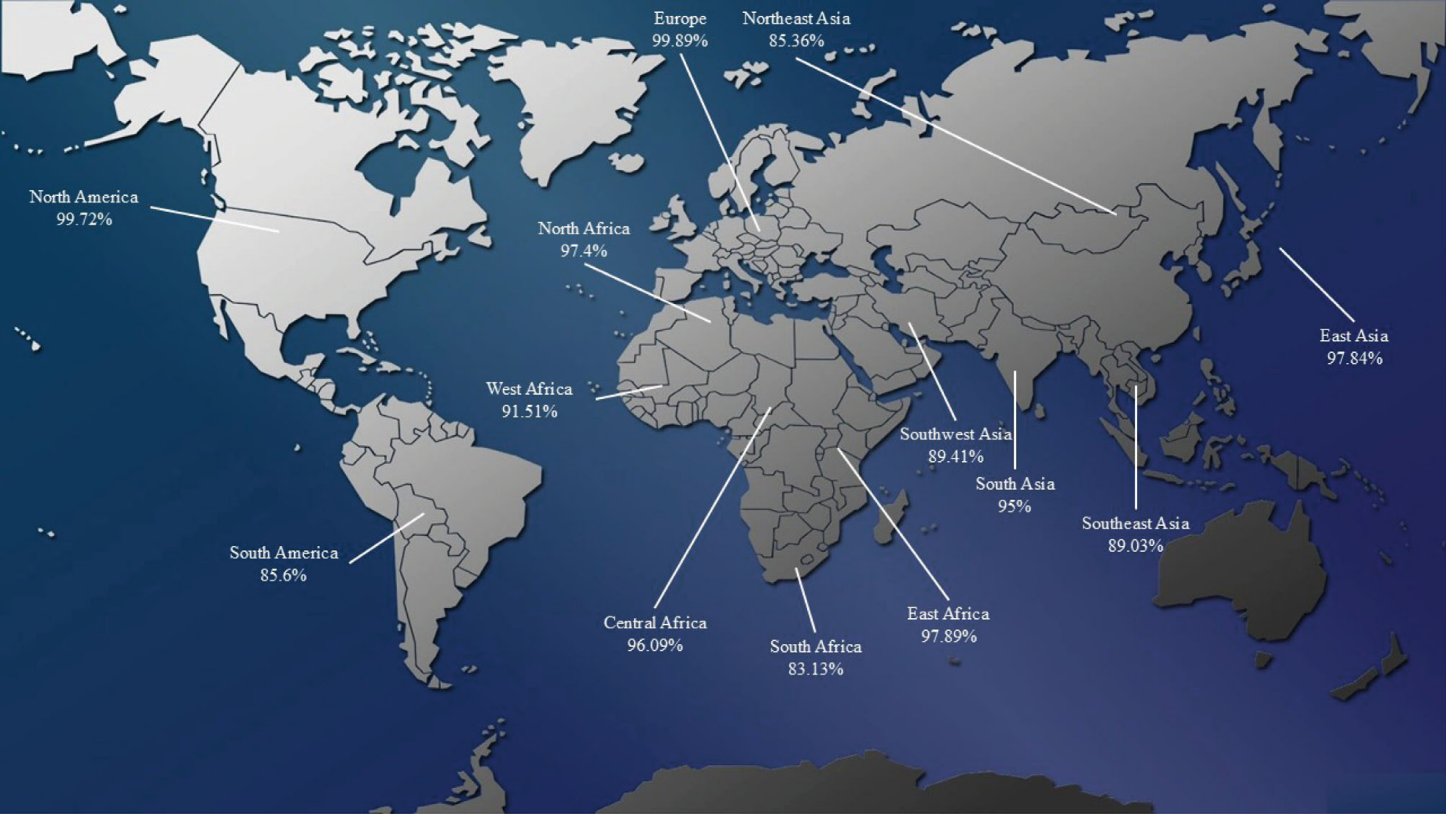
Population coverage of predicted epitopes across diverse geographic areas globally.

The population coverage analysis revealed that the predicted epitopes achieved a global coverage of 99.27%. Furthermore, Figure 1 illustrates the population coverage of the predicted epitopes in distinct regions worldwide.

### 3.4. Epitope‐MHC Molecular Docking

The docking process involving the analysis of interactions between epitopes and MHC molecules was conducted using the HDOCK server to assess the binding strength exhibited by these components. The outcomes of this analysis are meticulously available in Table 5.

**Table 5:** Result of epitope‐MHC molecular docking.

**MHC type**	**Pro type**	**HLA type**	**Epitope**	**Docking score**
MHC I	NOTCH1	HLA A 0201	DQIGEFQCI	−161.92
			CDLLTLTEY	−148.87
			CEWDGLDCA	−146.02
			CRAGHTGRR	−225.33
		HLA A 0301	DQIGEFQCI	−161.93
			CDLLTLTEY	−170.18
			CEWDGLDCA	−149.31
			CRAGHTGRR	−219.99
	NOTCH4	HLA A 0201	CQLRDFCSA	−184.92
			DLLNGFQCI	−160.04
			CSLGVPDPW	−171.55
			GQEGPRCEL	−153.74
		HLA A 0301	CQLRDFCSA	−178.71
			DLLNGFQCI	−169.66
			CSLGVPDPW	−170.07
			GQEGPRCEL	−151.12

MHC II	NOTCH1	HLA DRB1 0101	SSFHFLRELSRVLHT	−178.31
			RNSSFHFLRELSRVL	−223.37
			FHFLRELSRVLHTNV	−285.59
			QMIFPYYGREEELRK	−233.75
	NOTCH4	HLA DRB1 0101	ARAEEKLGGTRDPTY	−157.14
			PLLAVHPHAGTAPPA	−135.96
			TYQERAAPQTQPLGK	−239.08
			PWDPGLLLRFLAAMA	−236.83

### 3.5. Vaccine Construction

**Figure 2:**
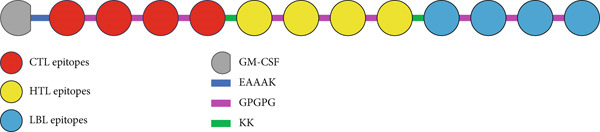
Schematic diagram showing the location of each vaccination component.

The vaccine is created by conjugating selected epitopes using appropriate linkers, and an adjuvant, GM‐CSF, is added at the C‐terminal of the vaccine. In Figure 2, a schematic diagram showing the location of each vaccination component displays the ultimate configuration of the vaccine.

### 3.6. Analysis of Physicochemical Properties and Posttranslational Modifications

#### 3.6.1. Evaluating the Physicochemical Properties, Allergenicity, Toxicity, and Antigenicity of the Designed Vaccine

Based on the findings from VaxiJen 2.0, AllerCatPro 2.0, and ToxinPred servers, the constructed vaccine showed acceptable levels of toxicity and allergenicity. It demonstrated a positive antigenicity value of 0.7017. Furthermore, the ProtParam server analysis revealed that the developed vaccine is a protein composed of 577 amino acids. The compound possesses a molecular weight of 60,670.57 and a pI value of 6.19, which is especially advantageous in the purification process. The estimated half‐life in human reticulocytes was 30 h in an in vitro setting, whereas yeast and *E. coli* had half‐lives of over 20 and 10 h, respectively. The instability index and aliphatic index were 39.70 and 62.10, respectively. Proteins must possess an instability index below 40 to be categorized as stable [77]. Hence, the developed vaccine exhibits robustness. The vaccine′s grand average of hydropathicity (GRAVY) was −0.471, indicating that the vaccination is predominantly hydrophilic (Table 6).

**Table 6:** Physicochemical characteristics of the designed vaccine.

**Characteristic**		**Amount**
Chemical composition	Atomic composition	
	C	2662
	H	4122
	N	772
	O	796
	S	31
	Total number of atoms	8383
	Formula	C_2662_H_4122_N_772_O_796_S_31_

Protein properties	Number of amino acids	577
	Molecular weight	60,670.57
	Theoretical pI	6.19
	Number of negatively charged residues (Asp + Glu)	58
	Number of positively charged residues (Arg + Lys)	52

Protein descriptors	Instability index	39.70
	Aliphatic index	62.10
	Grand average of hydropathicity (GRAVY)	−0.471
	Antigenicity	0.7017
	Allergenicity	Probable not allergen

#### 3.6.2. Evaluating Vaccine Posttranslational Modifications

The vaccine was analyzed to predict occurrences of five prevalent posttranslational modifications, namely, phosphorylation, N‐glycosylation, myristoylation, acetylation, and GPI modification sites. The results are shown in Table 7. The NetNGlyc‐1.0 server identified two glycosylation sites, and the NetPhos‐3.1 server detected 45 phosphorylation sites. According to the predictions made by three other servers, NetAcet‐1.0, Big‐PI/GPI Animals, and MyrPS/NMT, the construction showed no posttranslational modifications.

**Table 7:** Posttransitional modifications of the constructed vaccine.

**Posttranslational modification**	**Result**		
Phosphorylation	**Amino acid**	**Sites**	
	Ser	25	
	Thr	18	
	Tyr	2	
	Total	45	

N‐glycosylation	**Position**	**Potential**	**N-Glyc result**
	44	NLSR	0.7672
	54	NETV	0.7169

Acetylation	No significant N‐terminal acetylation sites		

Myristoylation	No myristoylation site		

Glycosylphosphatidylinositol (GPI) modification site	No potential GPI‐modification site		

### 3.7. Analysis of Secondary Structure

**Figure 3:**
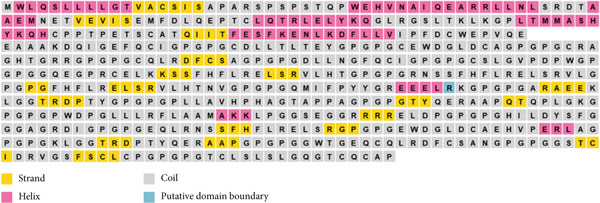
Secondary structure analysis of the constructed vaccine.

Using the PSIPRED V4.0 algorithm, the secondary structure of the vaccine protein was predicted, as shown in Figure 3. The analysis indicated that the structure includes 12.6% helix, 11.4% strand, and 0.17% putative domain boundary, with the remainder forming a coil conformation. This structural breakdown offers valuable insights into the protein′s flexibility and stability, essential factors in optimizing vaccine design and effectiveness.

### 3.8. Computational Modeling and Assessment of the Vaccine Tertiary Structure

The vaccine′s tertiary structure was predicted using the ROBETTA server and then refined using the GalaxyRefine server. Out of the five models provided by the GalaxyRefine server, the most optimal model was selected for further steps based on the findings obtained from ProCheck, ERRAT, Verify3D, and ProSa‐Web. The 3D structure of the vaccine was visualized using Chimera 1.171.1 (Figure 4a). The Ramachandran plot generated by the ProCheck server indicated that 90.1% of residues occupied the most favored regions, 7.2% were in additionally allowed regions, and 2.7% were in other regions, affirming the dynamic nature of the vaccine′s structure (Figure 4b). The ERRAT estimated a quality factor of 85.05. Verify3D showed that 90.64% of the residues have an average 3D‐1D score greater than or equal to 0.1 (Figure 4c). Moreover, the ProSa‐Web analysis yielded a Z score of −9.32 (Figure 4d).

Figure 4(a) Tertiary structure of the constructed vaccine. (b) Ramachandran plot result of ProCheck server. (c) Verify3D plot. (d) E–Z plot result of ProSa‐Web server.

**Figure:**
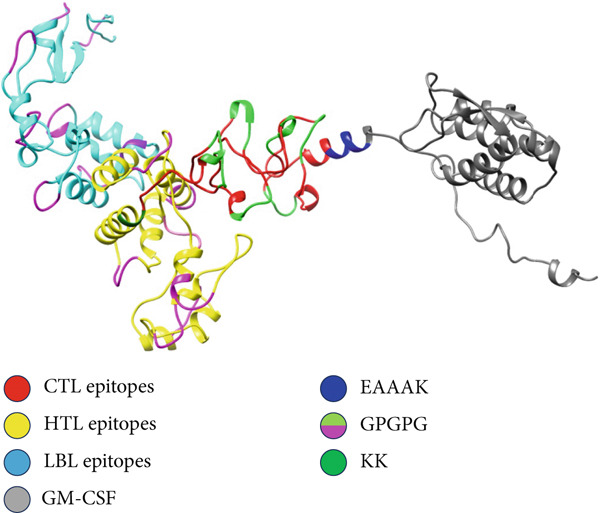
(a)

**Figure:**
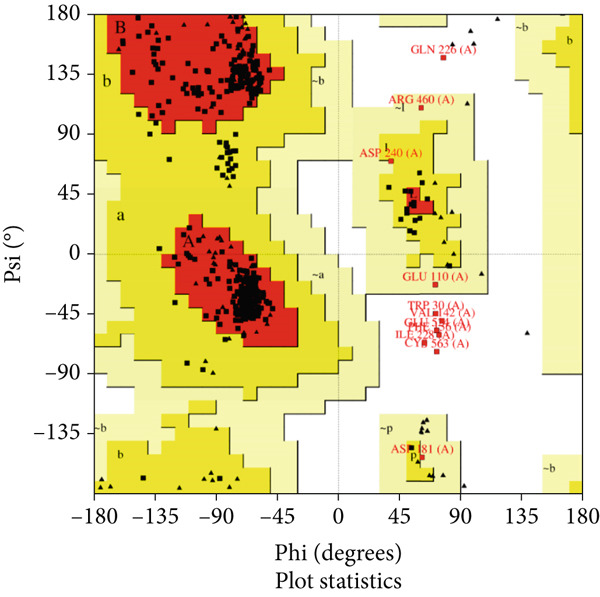
(b)

**Figure:**
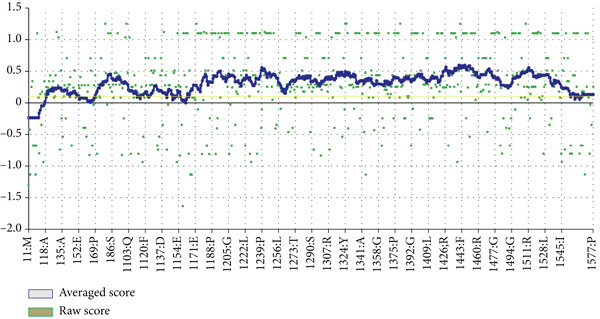
(c)

**Figure:**
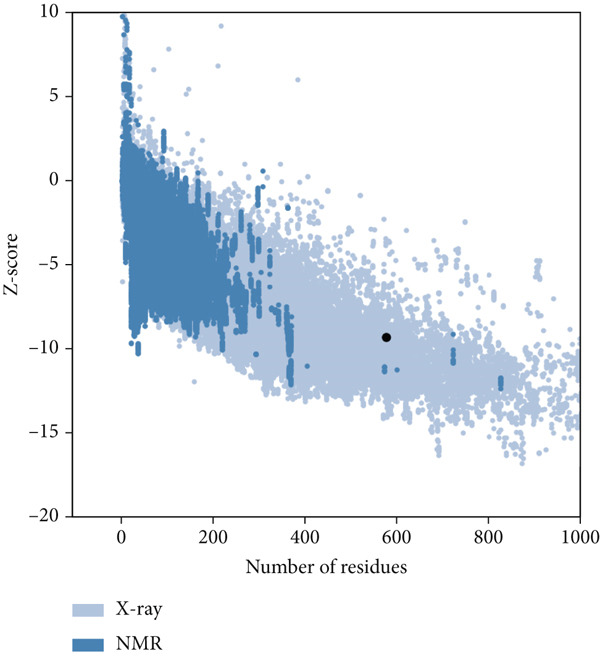
(d)

### 3.9. Analysis of Conformational B Cell Epitopes

**Figure 5:**
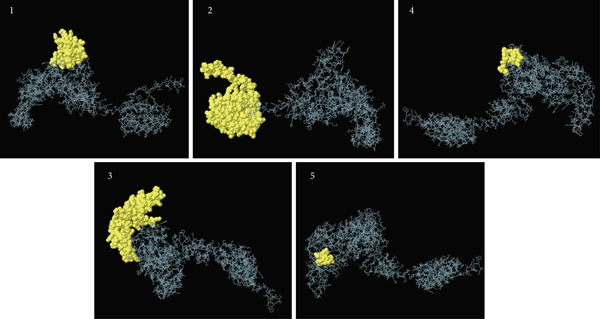
Conformational epitopes in 3D structure of the vaccine. The order of the numbers is according to Table 8.

The Ellipro server conducted a 3D structure analysis of the vaccine and identified five conformational epitopes. These epitopes have lengths ranging from 8 to 133 and were assigned scores between 0.54 and 0.744 (Table 8). Figure 5 displays the three‐dimensional configuration of these epitopes.

**Table 8:** Discontinuous epitopes of the constructed vaccine.

**No.**	**Residues**	**Number of residues**	**Score**
1	A:T273, A:G274, A:P275, A:G276, A:P277, A:G278, A:N280, A:S281, A:S282, A:M320, A:I321, A:F322, A:P323, A:Y324, A:Y325, A:G326, A:R327, A:E328, A:E329, A:E330, A:L331, A:R332, A:K333, A:G334, A:P335, A:G336, A:P337, A:G338, A:A339, A:R340, A:A341, A:E342, A:E343, A:K344, A:L345, A:G346, A:G347, A:T348, A:R349, A:D350, A:P351, A:T352, A:Y353, A:G354, A:P355, A:G356, A:P357, A:G358, A:P359, A:L360, A:L361	51	0.744

2	A:M1, A:W2, A:L3, A:Q4, A:S5, A:L6, A:L7, A:L8, A:L9, A:G10, A:T11, A:V12, A:A13, A:C14, A:S15, A:I16, A:S17, A:A18, A:P19, A:A20, A:R21, A:S22, A:P23, A:S24, A:P25, A:S26, A:T27, A:Q28, A:P29, A:W30, A:E31, A:H32, A:V33, A:N34, A:A35, A:I36, A:Q37, A:E38, A:A39, A:R40, A:R41, A:L42, A:L43, A:N44, A:L45, A:S46, A:R47, A:D48, A:T49, A:A50, A:A51, A:E52, A:M53, A:N54, A:E55, A:T56, A:V57, A:E58, A:V59, A:I60, A:S61, A:E62, A:M63, A:E68, A:P69, A:T70, A:C71, A:L72, A:Q73, A:T74, A:R75, A:L76, A:E77, A:L78, A:Y79, A:K80, A:Q81, A:G82, A:L83, A:R84, A:G85, A:S86, A:L87, A:T88, A:K89, A:L90, A:K91, A:G92, A:P93, A:L94, A:T95, A:M96, A:M97, A:A98, A:S99, A:H100, A:Y101, A:K102, A:Q103, A:H104, A:C105, A:P106, A:P107, A:T108, A:P109, A:E110, A:T111, A:S112, A:C113, A:A114, A:T115, A:Q116, A:I117, A:I118, A:T119, A:F120, A:E121, A:S122, A:F123, A:K124, A:E125, A:N126, A:L127, A:K128, A:D129, A:F130, A:L131, A:L132, A:I134, A:P135, A:F136, A:D137, A:C138	133	0.739

3	A:E428, A:L429, A:D430, A:P431, A:G432, A:P433, A:G434, A:R472, A:G473, A:P474, A:G475, A:P476, A:G477, A:W479, A:G494, A:P495, A:G496, A:P497, A:G498, A:K499, A:L500, A:G501, A:G502, A:T503, A:R504, A:D505, A:P506, A:T507, A:Y508, A:Q509, A:E510, A:R511, A:A512, A:A513, A:P514, A:G515, A:P516, A:G517, A:P518, A:G519, A:G520, A:W521, A:T522, A:G523, A:E524, A:Q525, A:C526, A:Q527, A:L528, A:R529, A:D530, A:F531, A:C532, A:S533, A:A534, A:N535, A:G536, A:P537, A:G538, A:P539, A:G540, A:G541, A:S542, A:T543, A:C544, A:I545, A:D546, A:R547, A:V548, A:G549, A:S550, A:F551, A:S552, A:C553, A:L554, A:C555, A:P556, A:G557, A:P558, A:G559, A:P560, A:G561, A:T562, A:C563, A:L564, A:S565, A:L566, A:S567, A:L568, A:G569, A:Q570, A:G571, A:T572, A:C573, A:Q574, A:C575, A:A576, A:P577	98	0.726

4	A:A370, A:P371, A:P372, A:A373, A:G374, A:P375, A:G376, A:P377, A:G378, A:T379, A:Q381	11	0.583

5	A:I451, A:G452, A:P453, A:G454, A:P455, A:G456, A:E457, A:Q458	8	0.54

### 3.10. Immune Simulation Analysis Result

The vaccination protocol involved three administrations spaced 28 days apart. Immunological responses exhibited significant escalation with each administration. Following the decline in antigen levels, immunoglobulin levels remained elevated. The level of IgM was higher than IgG, as shown in Figure 6a. This implies the vaccine′s capacity to elicit prolonged immune memory and sustain a persistent immune response. Furthermore, there was an observed rise in the number of B cells after every injection, and their population, especially the population of memory B cells, sustained elevated levels well beyond the administration of the final vaccine dosage (Figure 6b, and 6c). Moreover, there was a substantial increase in the population of memory T cells and cytotoxic T lymphocytes (CTLs), indicating successful memory cell generation (Figures 6d, 6e, 6f, and 6g). Additionally, interleukin‐2 (IL‐2) and INF‐G increased following each injection (Figure 6i). Details regarding the populations of dendritic cells, macrophages, and natural killer (NK) cells are depicted in Figures 6h, 6j, and 6k.

Figure 6Immune simulation result of C‐ImmSim server. (a) The immunoglobulins and the immunocomplexes. (b) B lymphocytes: total count, memory cells, and subdivided into isotypes IgM, IgG1, and IgG2. (c) B lymphocyte population per entity state. (d) CD4 T‐helper lymphocyte count subdivided per entity state. (e) CD4 T‐helper lymphocyte count. (f) CD8 T‐cytotoxic lymphocytes count per entity state. (g) CD8 T‐cytotoxic lymphocytes count. (h) Natural killer cells (total count). (i) Concentration of cytokines and interleukins. (j) Macrophages: total count, internalized, presenting on MHC Class II, active and resting macrophages. (k) Dendritic cells.

**Figure:**
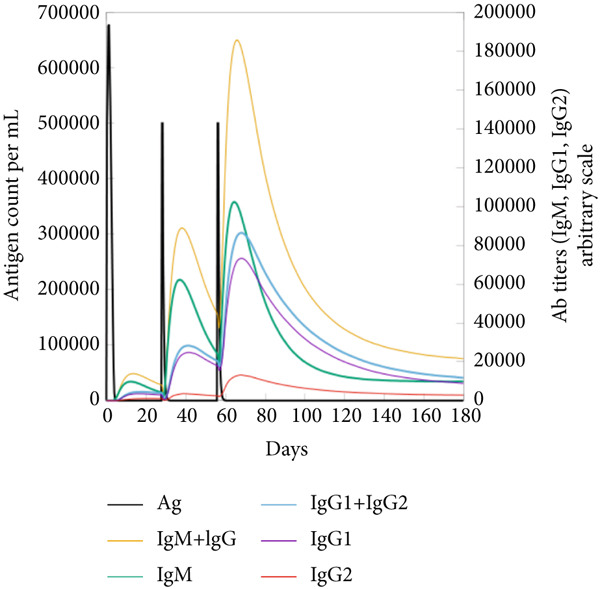
(a)

**Figure:**
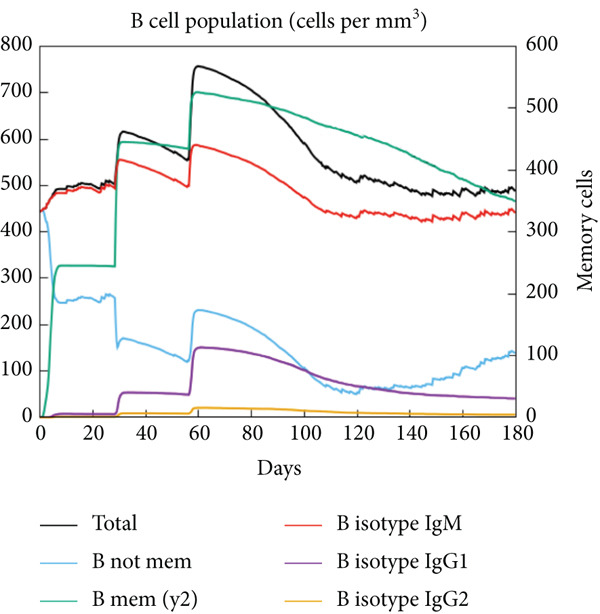
(b)

**Figure:**
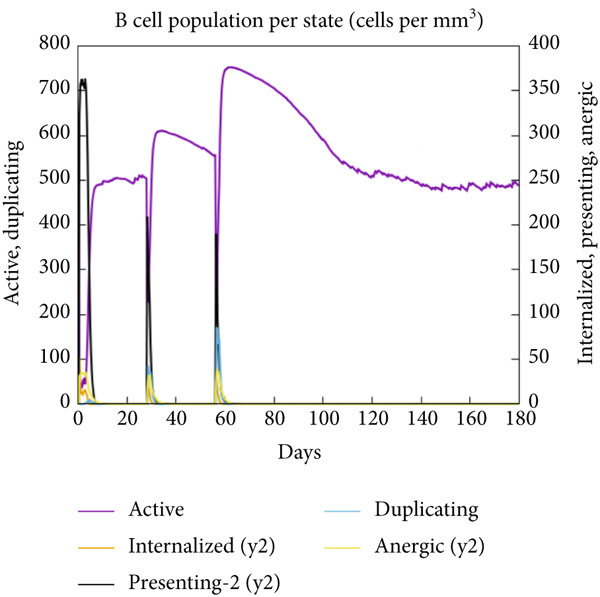
(c)

**Figure:**
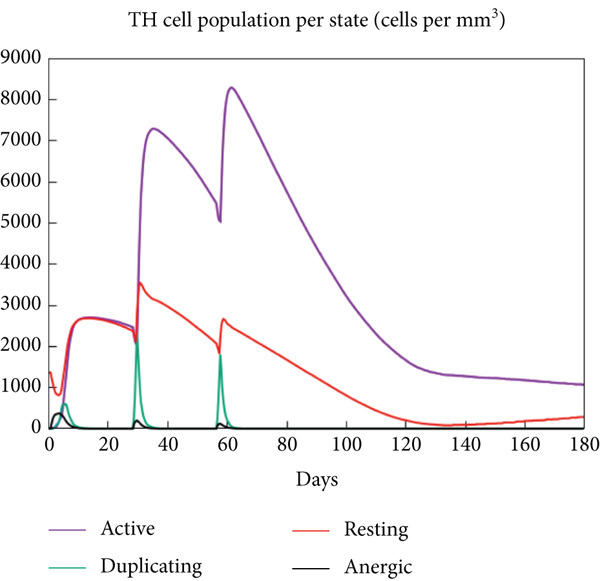
(d)

**Figure:**
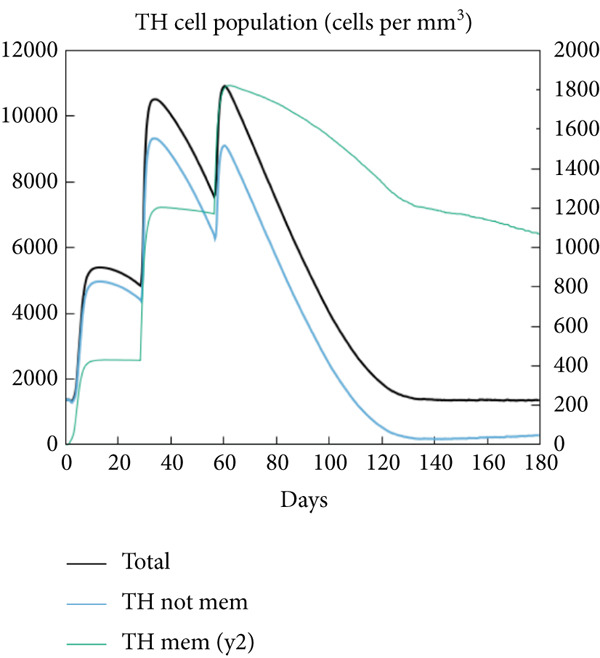
(e)

**Figure:**
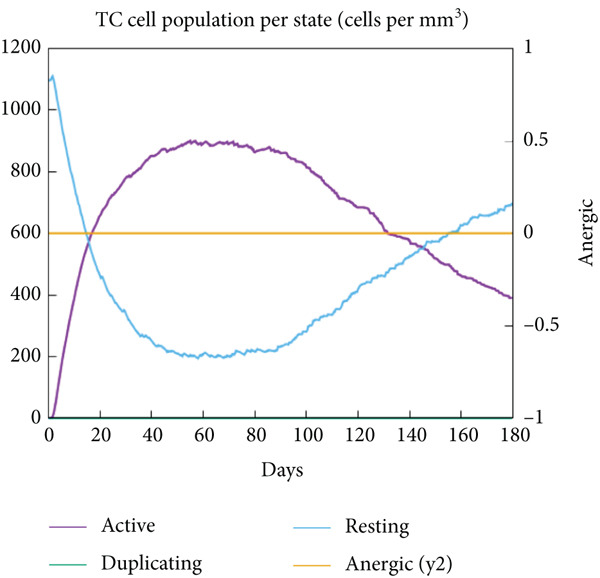
(f)

**Figure:**
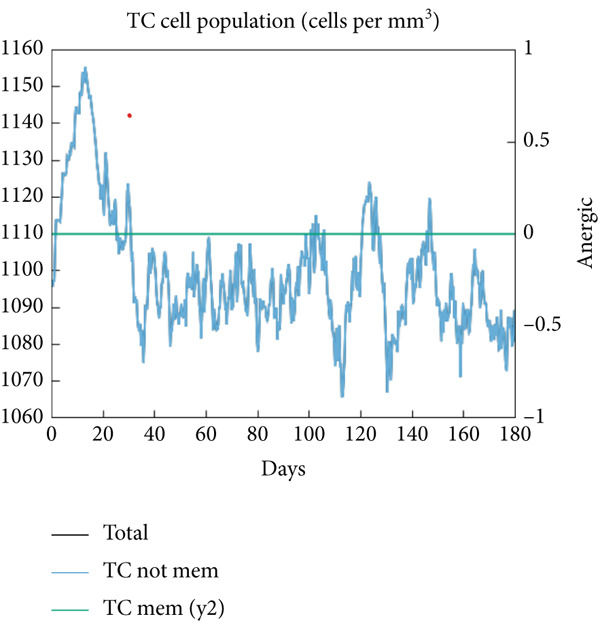
(g)

**Figure:**
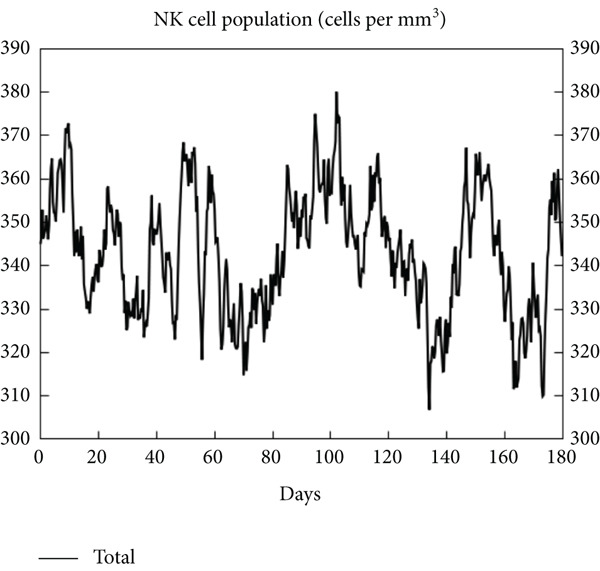
(h)

**Figure:**
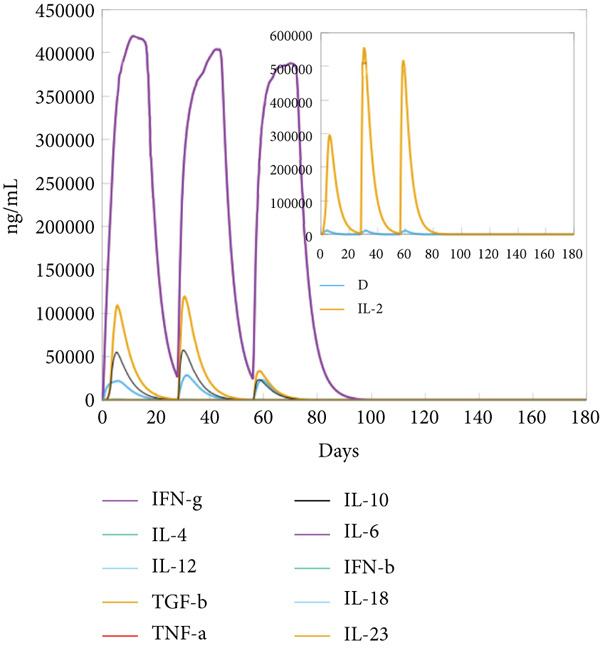
(i)

**Figure:**
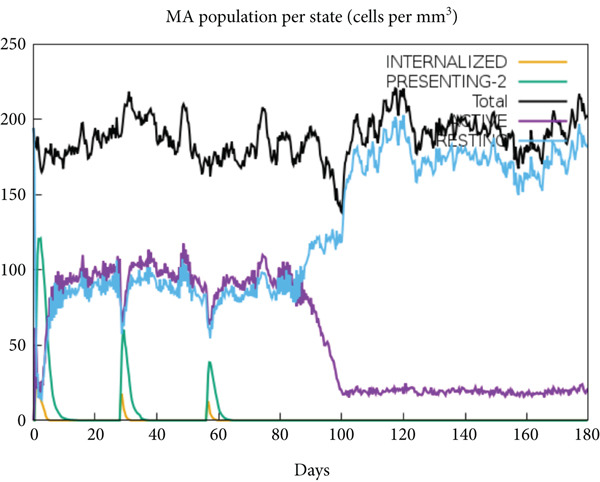
(j)

**Figure:**
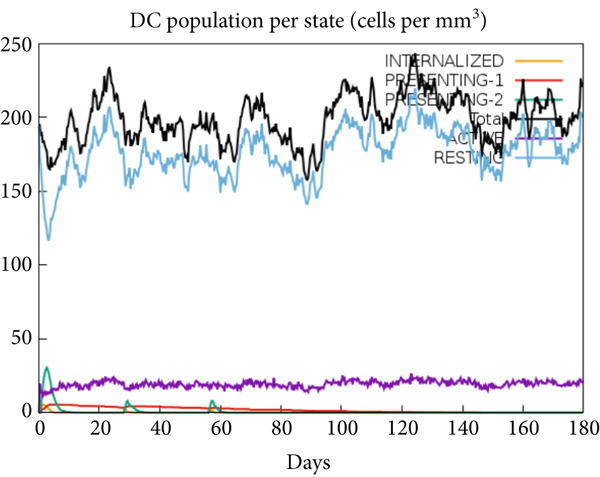
(k)

### 3.11. Molecular Docking Analysis

The findings from the docking analysis of the vaccine′s 3D structure with HLA A 0201, HLA A 0301, HLA DRB1 0101, TLR4, and TLR7, performed via the HDOCK server, are presented in Table 9. The best models were selected, and their binding affinities are presented. Additionally, the 3D structure of the docking results is displayed in Figure 7 using Chimera 1.171.1. Also, the PRODIGY server calculated *Δ*G and Kd for each docked structure, as shown in Table 9. The vaccine demonstrates a strong binding affinity across receptors, with the highest docking score of −317.05 for TLR7 and the most favorable *Δ*G of −15.5 kcal/mol for TLR4. Notably, TLR4 shows the lowest Kd (4.5e‐12 M), suggesting its significant role in immune response activation compared with HLA interactions.

**Table 9:** Analysis results of docked structures.

**Receptor**	**Ligand**	**Docking score**	***Δ*G**	**Kd**
HLA A 0201	Vaccine	−267.49	−13.1	2.3e‐10
HLA A 0301	Vaccine	−283.63	−14.0	5.2e‐11
HLA DRB1 0101	Vaccine	−288.97	−14.1	4.7e‐11
TLR 4	Vaccine	−277.87	−15.5	4.5e‐12
TLR 7	Vaccine	−317.05	−12.6	5.7e‐10

Figure 73D structures of docking results. (a) Docking of vaccine with HLA A 0201. (b) Docking of vaccine with HLA A 0301 (c) Docking of vaccine with HLA DRB1 0101. (d) Docking of vaccine with TLR 4. (e) Docking of vaccine with TLR 7. The yellow regions represent the vaccine structure, whereas the rainbow‐colored regions indicate the receptor structure.

**Figure:**
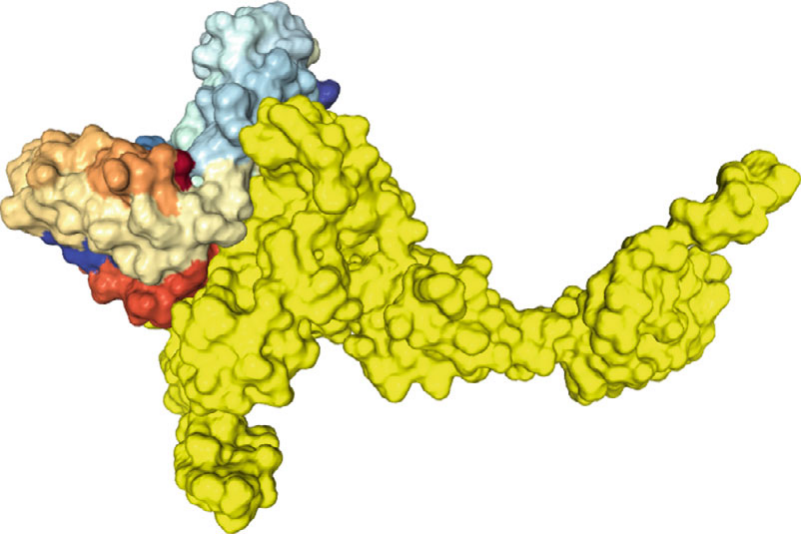
(a)

**Figure:**
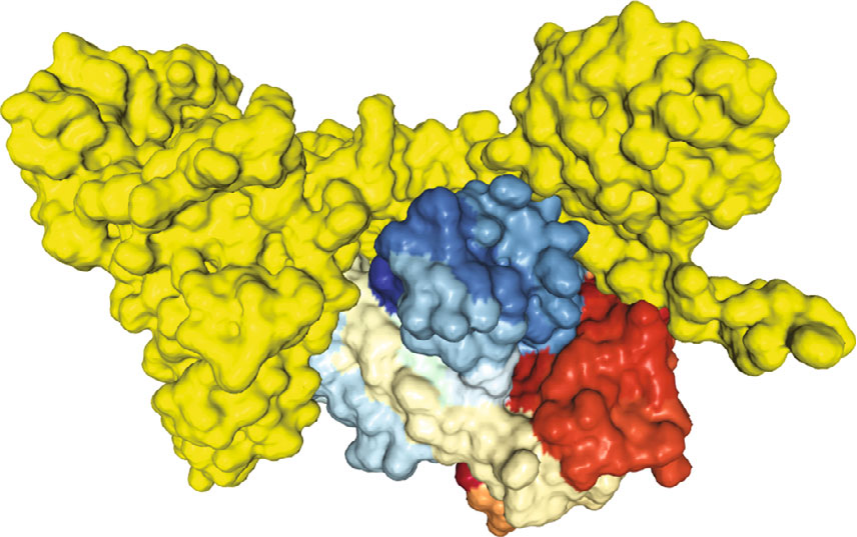
(b)

**Figure:**
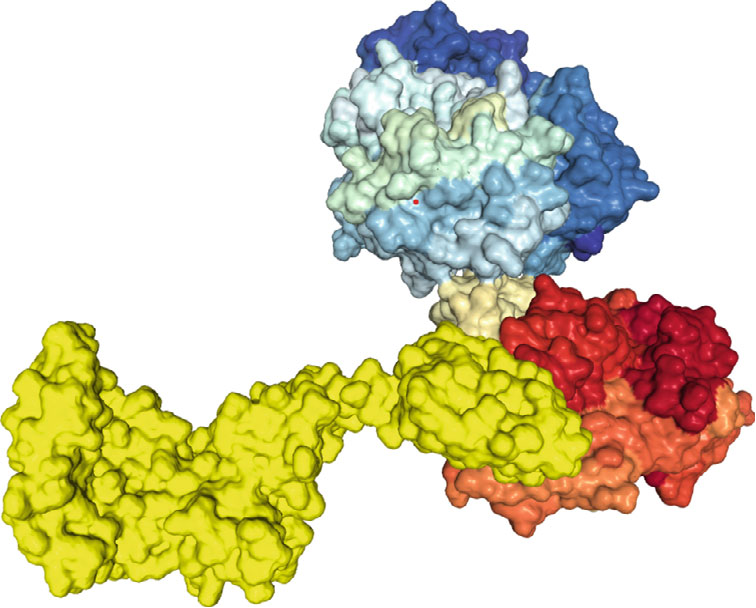
(c)

**Figure:**
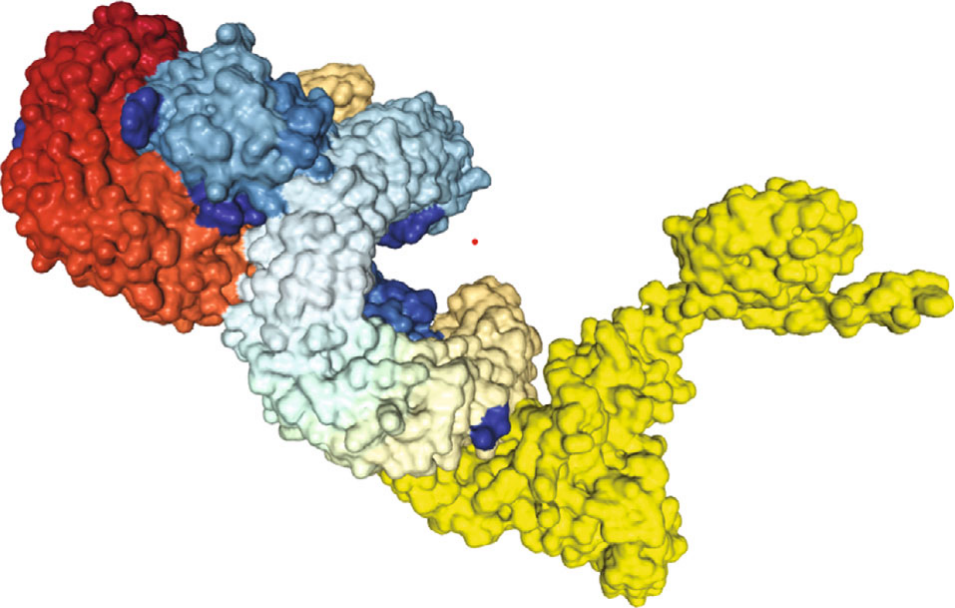
(d)

**Figure:**
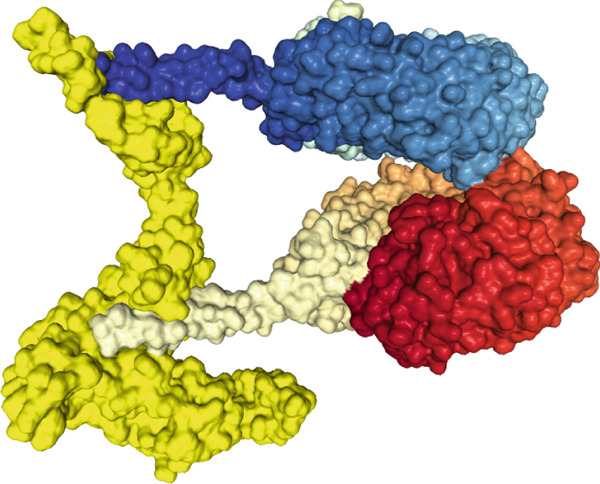
(e)

PDBsum analysis revealed that the docked vaccine complex with MHC I, MHC II, TLR4, and TLR7 exhibits key interactions. The types and quantities of each interaction are detailed in Table 10. Figure 8 details specific amino acid pairs where each receptor chain interacts with the vaccine, highlighting critical residues involved in H‐bonding, salt bridges, and nonbonded contacts. This visualization provides insight into the binding interfaces and interactions.

**Table 10:** Summary of interactions between the docked vaccine complex and receptors MHC I, MHC II, TLR4, and TLR7.

**Receptor**	**Chain ID**	**No. of interface residues**	**No. of salt bridges**	**No. of disulfide bonds**	**No. of hydrogen bonds**	**No. of nonbonded contacts**
TLR 7	A	10:15	—	—	1	102
	B	10:13	—	—	—	88

TLR 4	B	24:25	4	—	3	224
	D	6:8	—	—	1	32

MHC II	A	10:13	3	—	3	112
	B	17:15	—	—	3	124
	D	4:4	—	—	—	17
	E	4:4	—	—	—	23

MHC I (A02)	A	17:22	2	—	4	120
	B	8:9	3	—	3	34

MHC I (A03)	A	17:15	2	—	2	86
	B	10:12	—	—	2	104

Figure 8Schematic representation of amino acid interactions between the docked vaccine and each receptor chain within the complex: (a) (HLA A03), (b) (HLA A02), (c) (HLA DRB1), (d) (TLR4), and (e) (TLR7).

**Figure:**
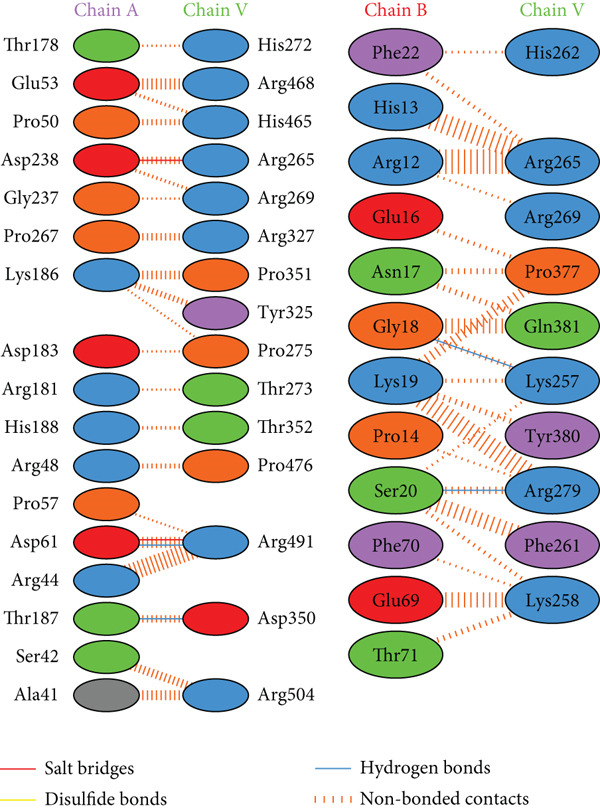
(a)

**Figure:**
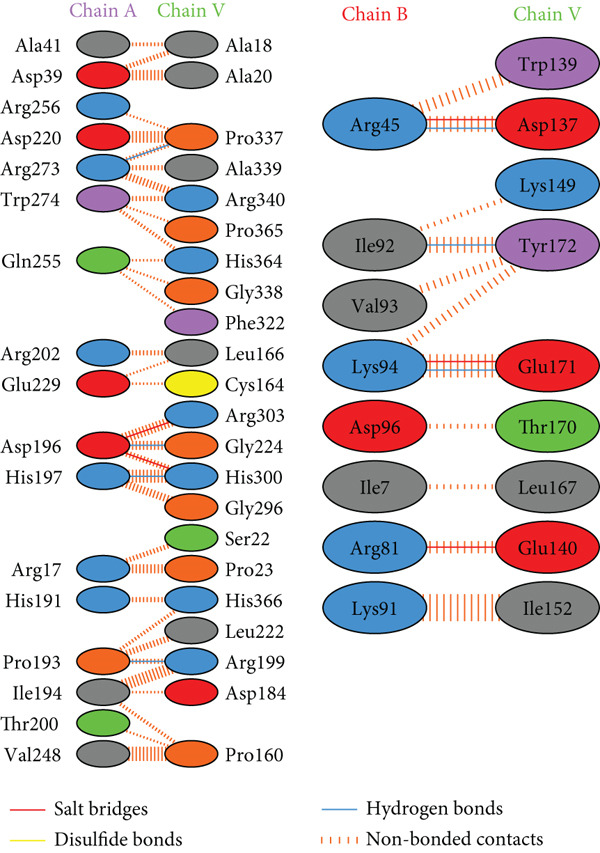
(b)

**Figure:**
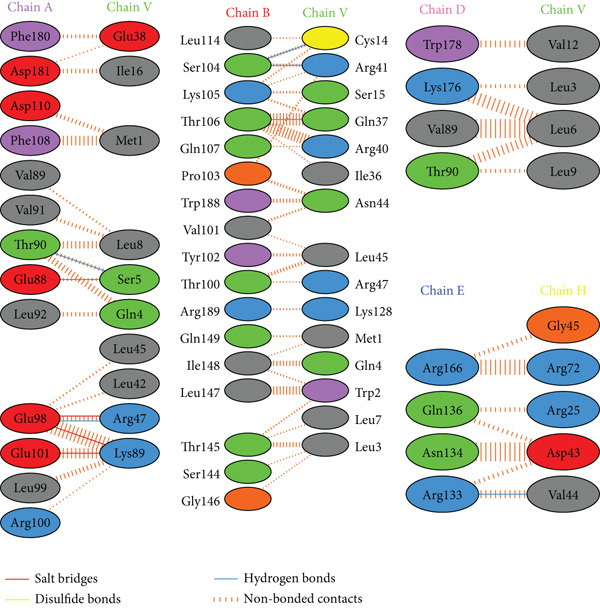
(c)

**Figure:**
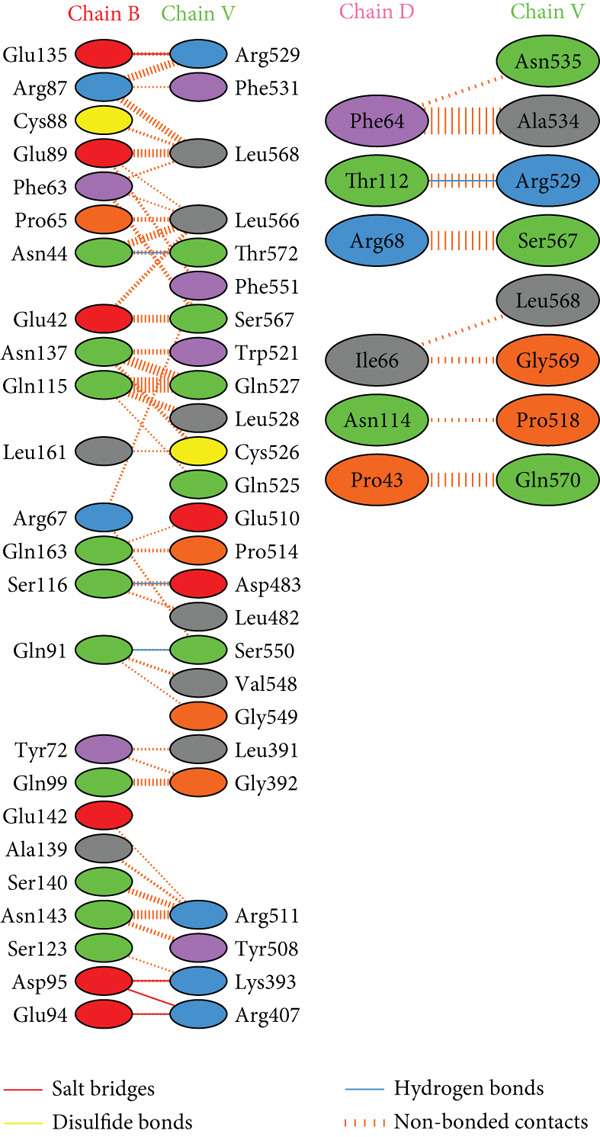
(d)

**Figure:**
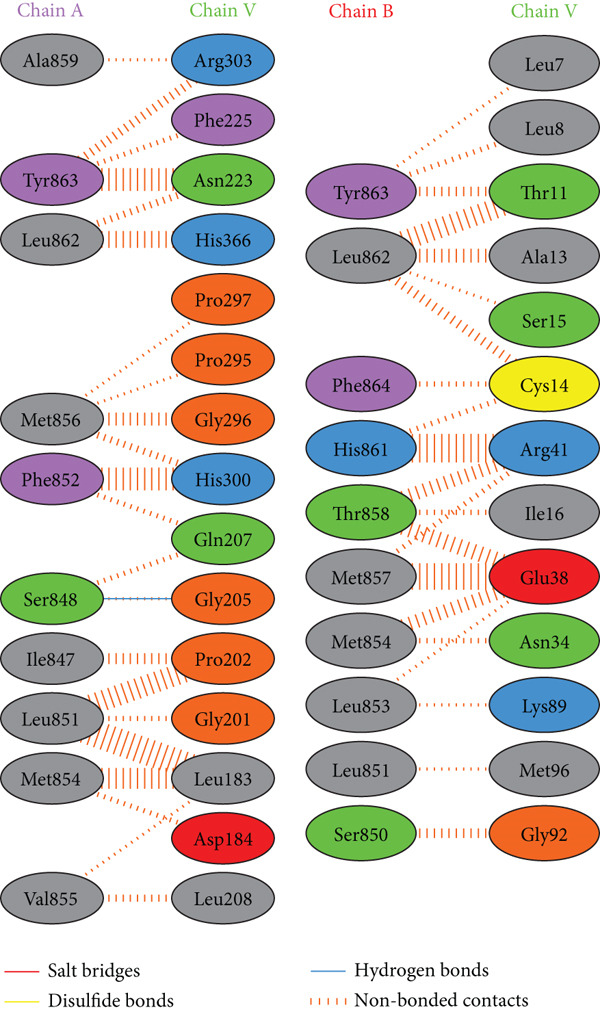
(e)

The difference in interaction counts across chains highlights unique binding affinities and structural contributions of each receptor within the docked vaccine complex. TLR4 (Chain A), with its four salt bridges and three H‐bonds, and MHC II (Chain A), with three salt bridges and three H‐bonds, show pronounced electrostatic and H‐bond stabilization, which may enhance receptor affinity. MHC I (A02) Chain A, with two salt bridges and four H‐bonds, and Chain B, with three of each interaction type, suggests balanced electrostatic and H‐bond contributions for stability. In contrast, MHC I (A03) Chain A, which contains two salt bridges and two H‐bonds, reflects moderate stabilizing interactions.

Nonbonded contacts are highest in TLR4 (Chain B) and MHC II (Chain B), suggesting that van der Waals forces play a significant role in binding for these regions, potentially indicating stronger stabilization. Disulfide bonds are limited and occur selectively across chains, conferring specific covalent stabilization within the complex. These distinct patterns in interactions—salt bridges for electrostatic stability, H‐bonds for binding affinity, and nonbonded contacts for structural cohesion—underscore the essential roles of each receptor in maintaining the vaccine‐receptor complex′s structural integrity and potentially enhancing its efficacy.

### 3.12. MD Analysis

#### 3.12.1. RMSD

**Figure 9:**
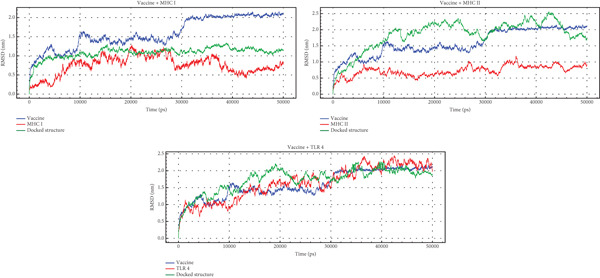
The RMSD profiles for the docked complexes (green) alongside the individual multi‐epitope vaccine (blue) and immune receptors MHC I, MHC II, and TLR4 (red) from the MD simulation analysis.

RMSD analysis provides insights into the dynamic behavior of the vaccine and its docked structure in complex with TLR4, MHC I, and MHC II. RMSD quantifies the average deviation of a structure from a reference point, in this case, the initial docked conformation. Across all three systems, the docked structure exhibits a progressive increase in RMSD from 0 nm, indicating a deviation from the starting model. Specifically, the docked structure in the Vaccine + MHC II system reaches a higher RMSD plateau of approximately 2 nm compared with the Vaccine + MHC I system, which plateaus around 1.2 nm, suggesting a greater degree of conformational exploration in the presence of MHC II. Comparing the vaccine′s RMSD with that of the docked structure, the vaccine demonstrates distinct interaction patterns with each receptor. With TLR4 and MHC II, the vaccine achieves relatively stable complexes following initial adjustments, fluctuating around 1.5–2 nm after 20,000 ps. In contrast, the interaction with MHC I involves more pronounced conformational shifts, as evidenced by the stepwise RMSD increase, reaching approximately 2 nm after 30,000 ps. These findings are visualized in Figure 9.

#### 3.12.2. RMSF

**Figure 10:**
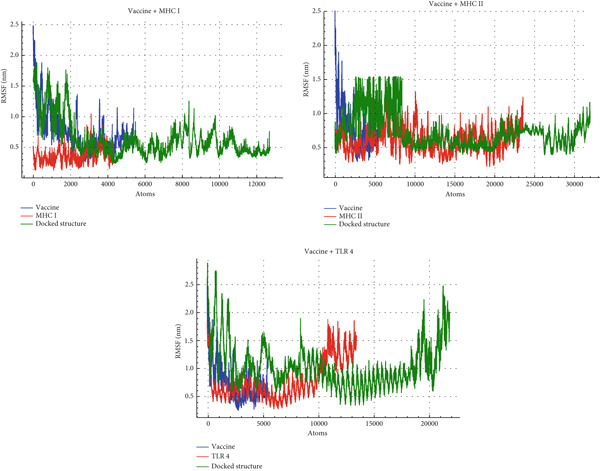
The RMSF profiles for the docked complexes (green) alongside the individual multi‐epitope vaccine (blue) and immune receptors MHC I, MHC II, and TLR4 (red) from the MD simulation analysis.

RMSF measures the average fluctuation of each residue in a protein during a MD simulation, typically in Angstroms (Å), revealing residue‐level dynamics and flexibility. Across all three systems (Vaccine + MHC I, MHC II, and TLR4), the docked structure consistently exhibits the highest flexibility, with RMSF values peaking around 4 Å, indicating that complex formation induces conformational changes. In the Vaccine + MHC I system, MHC I remains relatively rigid (below 2 Å), whereas the vaccine shows moderate flexibility, reaching up to 3 Å. The Vaccine + MHC II system displays similar fluctuation patterns for both MHC II and the vaccine, ranging between 1 Å and 3 Å. Conversely, in the Vaccine + TLR4 system, TLR4 exhibits moderate fluctuations (1–3 Å), while the vaccine shows lower fluctuations (mostly below 2 Å). These variations in RMSF profiles suggest differences in binding affinity, complex flexibility, and overall interaction dynamics between the vaccine and each receptor. The vaccine′s flexibility varies depending on the receptor, with MHC I inducing the most significant fluctuations. These dynamics are clearly illustrated in Figure 10.

#### 3.12.3. Rg

**Figure 11:**
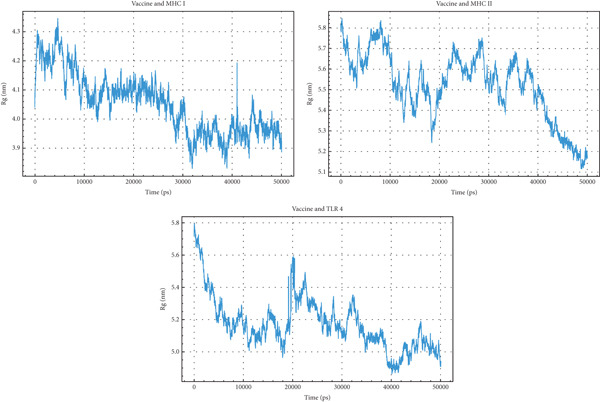
Radius of gyration (Rg) analysis of the vaccine in complex with MHC I, MHC II, and TLR4 over a 50,000 ps simulation.

Rg measures the compactness of a protein, indicating its overall dimensions in nanometers (nm). In the Vaccine + MHC I complex, Rg fluctuates between 4.0 and 4.3 nm initially, then decreases to 3.9–4.1 nm after 30,000 ps, suggesting a slight compaction. The Vaccine + MHC II complex starts with a higher Rg around 5.8 nm, gradually decreasing to approximately 5.2 nm by the end of the simulation, indicating a significant compaction. Similarly, the Vaccine + TLR4 complex shows a decrease in Rg from 5.8 nm to around 5.0 nm, also suggesting compaction. Comparing the three, the Vaccine + MHC I complex exhibits the smallest Rg values and the least compaction, implying a more stable and compact initial conformation. Vaccine complexes with MHC II and TLR4 start with larger, more extended conformations that become more compact during the simulation. The greater decrease in Rg for MHC II and TLR4 suggests that the vaccine undergoes more significant conformational changes when interacting with these receptors compared with MHC I. This implies different binding mechanisms and induced fit processes for each complex (Figure 11).

#### 3.12.4. SASA

**Figure 12:**
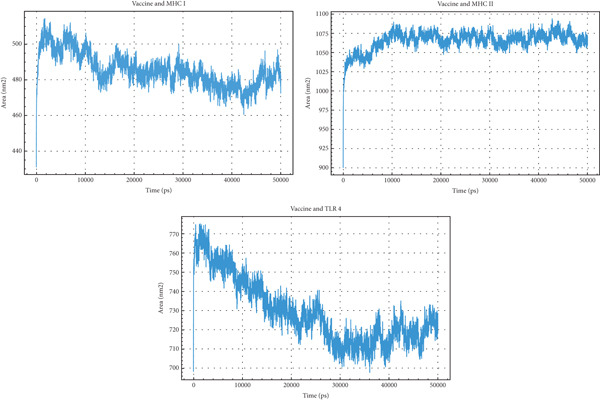
Solvent accessible surface area (SASA) analysis of the vaccine complexed with MHC I, MHC II, and TLR4.

SASA measures the area of a protein that is accessible to solvent molecules, providing insights into protein folding and interactions, expressed in nm^2^. The Vaccine + MHC I complex exhibits a SASA that rises rapidly to approximately 500 nm^2^ and then fluctuates between 460 and 500 nm^2^ throughout the simulation, showing a slight decrease towards the end. The Vaccine + MHC II complex shows a SASA increase to roughly 1075 nm^2^, maintaining a relatively stable range between 1050 and 1075 nm^2^ with minor fluctuations. The Vaccine + TLR4 complex SASA increases to about 770 nm^2^, then decreases and fluctuates between 700 and 740 nm^2^ after 20,000 ps. Comparing the three, the Vaccine + MHC II complex exhibits the highest SASA, suggesting a more extended and solvent‐exposed conformation. The Vaccine + TLR4 complex shows a significant decrease in SASA over time, indicating a conformational change leading to reduced solvent accessibility. The Vaccine + MHC I complex has the lowest SASA, implying a more compact and less solvent‐exposed structure. These differences suggest distinct interaction mechanisms and conformational adaptations of the vaccine when binding to different receptors (Figure 12).

#### 3.12.5. H‐Bond Analysis

**Figure 13:**
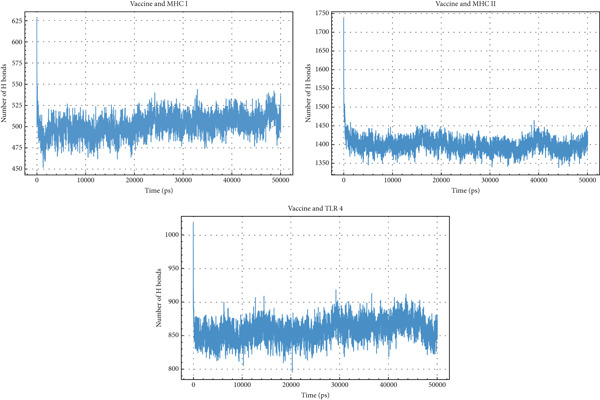
H‐bonds analysis of vaccine interactions with MHC I, MHC II, and TLR4.

H‐bonds are crucial for stabilizing protein–protein interactions. The Vaccine + MHC I complex shows a rapid initial increase to around 625 H‐bonds, quickly stabilizing and fluctuating between 475 and 525 H‐bonds throughout the simulation. The Vaccine + MHC II complex exhibits a much higher number of H‐bonds, starting around 1750, then decreasing to fluctuate between 1350 and 1450 H‐bonds. The Vaccine + TLR4 complex displays an initial peak of around 1025 H‐bonds, which then decreases to fluctuate between 825 and 900 H‐bonds. Comparing the three, the Vaccine + MHC II complex consistently forms the highest number of H‐bonds, indicating a stronger and more stable interaction compared with the other two. The Vaccine + TLR4 complex forms an intermediate number of H‐bonds, whereas the Vaccine + MHC I complex forms the fewest. These differences in H‐bond numbers suggest varying binding affinities and interaction strengths between the vaccine and each receptor, with MHC II exhibiting the strongest interaction, followed by TLR4, and then MHC I (Figure 13).

The second and third repetition diagrams are provided in the supporting information file.

## 4. Discussion

TNBC, an aggressive type of breast cancer, faces challenges in treatment due to its lack of targetable receptors. Traditional treatments are the mainstay, involving surgery, chemotherapy, and radiotherapy for early‐stage interventions. There is a pressing need for innovative therapies, such as cancer immunotherapy, to enhance clinical management and cure rates. Tumor vaccination, offering high specificity and minimal adverse effects compared to radiotherapy and chemotherapy, holds promise. Nevertheless, technological constraints have hindered satisfactory success rates [78]. Therefore, finding the best target in TNBC through in silico studies is vital.

In TNBC, various in silico studies have been carried out on multiple targets. In the study of Parvizpour et al., peptide vaccines against the NY‐ESO‐1 and MAGE‐A family antigens were investigated [79]. In another study by Krishnamoorthy and Karuppasamy, they designed a peptide‐based vaccine against the MZF‐1 antigen [80]. Dariushnejad et al. designed recombinant vaccines against CEA, MTDH, and MUC‐1 proteins [81]. Since the in silico study of NOTCH1 and NOTCH4 had yet to be investigated in TNBC, this aim was investigated in this study.

The Notch signaling pathway is essential for the development and progression of TNBC. Notch receptors are critical to controlling the activity of tumor‐initiating cells and the development of TNBC. Furthermore, substantial evidence indicates that the Notch pathway is crucial in preserving and growing breast cancer cells. The expression and activation of Notch receptors are intricately associated with the aggressive clinicopathological and biological features of breast cancer, such as invasiveness and resistance to treatment. These characteristics are particularly pertinent to the TNBC subtype [82].

An ideal vaccine has the least allergenicity and toxicity and the most antigenicity for the immune system. Indeed, an optimal vaccine should stimulate all facets of the immune system, encompassing the innate immune system, B cells, and T cells, to effectively target its intended objective while maintaining a high degree of selectivity [83]. Epitope prediction for MHC I and MHC II is crucial in vaccine design. It involves identifying small protein fragments that bind to these MHC molecules and trigger effective immune responses. Accurate prediction ensures optimal recognition by T cells, enabling the design of vaccines that induce robust immunity against infections and diseases [84–86].

The designed vaccine was protein‐based. EAAAK, KK linker, and GPGPG spacer were used in this vaccine. These linkers were selected for their roles in enhancing flexibility and supporting the proper folding of the vaccine construct, which is crucial to ensuring the protein′s functional domains are appropriately spaced for optimal functionality [87]. The EAAAK linker is a frequently used sequence in protein engineering to connect several protein domains or components, offering flexibility and retaining optimal folding and function [88]. The KK linker offers flexibility and creates spatial separation between functional domains or epitopes in protein constructs [89]. Adjuvants potentiate and prolong immune responses, without which effective cancer vaccines could hardly find their appeal. Significantly, this impact was shown to be due to the increased T cell activation through three main parameters: antigen presentation, co‐stimulation, and cytokine signaling. Different classes of adjuvants may impart these signals differently by enhancing antigen uptake, further cytokine production, and stimulating immune cells. Aside from pathogen‐derived molecules, very recently, it has been suggested that cytokines may represent an effective adjuvant. GM‐CSF has frequently been used in several cancer vaccine studies as a principal immunomodulatory molecule [90]. GM‐CSF acted as an adjuvant, providing enhanced potency and kinetics of immune responses, thus bringing most of the value into the vaccine development process [91, 92].

Analyses of the posttranslational modifications of the constructed vaccine and of predicting posttranslational modifications such as phosphorylation, N‐glycosylation, myristoylation, acetylation, and GPI modification sites were investigated in the present study. Myristoylation is essential for guiding and tethering proteins to membranes, serving as a critical lipid modification located at the N‐terminus of eukaryotic proteins. This modification holds significance in cellular regulation, signal transduction, translocation, and even apoptosis [59]. The GPI modification site is important in posttranslational modifications of eukaryotic proteins that are typically secreted and involve attaching such proteins to the plasma membranes [56]. These modifications should be considered when choosing a host for vaccine protein expression.

Molecular docking is a pivotal technique in drug discovery and pharmaceutical research, facilitating the prediction of how molecules interact and aiding in vaccine design [93]. Molecular docking results revealed that the crafted vaccine effectively engages with immune system molecules. Additionally, vaccine analysis indicated a sustained elevation in immunoglobulin levels, signifying the vaccine′s capacity to induce prolonged immune memory and maintain a persistent immune response. The increased presence of B cells, memory T cells, and CTLs further indicates the successful generation of memory cells. In summary, the outcomes of this study affirm the suitability of targeting NOTCH1 and NOTCH4 as a vaccine candidate against TNBC.

There are a series of limitations in vaccine design; epitope variability in target proteins, individual genetic variation, and immunogenicity prediction challenges make it difficult to identify universally effective epitopes, limited knowledge of antigenic targets, adjuvant selection, posttranslational modifications, population‐specific responses, regulatory hurdles, manufacturing complexity, and long‐term immune memory also pose challenges. Although there are limitations, in silico studies should be performed to obtain valuable data for the design of in vitro and in vivo studies.

## 5. Conclusion

The designed protein vaccine targeting NOTCH1 and NOTCH4 antigens demonstrates promising potential against TNBC. The findings, including the vaccine′s high antigenicity, strong binding affinity to MHC molecules and TLRs, and predicted ability to stimulate a robust and prolonged immune response, underscore the promise of this vaccine candidate. However, the limitations inherent in vaccine design, such as epitope variability and individual genetic variations, exist. Therefore, although the in silico analysis provides crucial data and strong support for the vaccine′s potential, further validation through in vitro and in vivo studies is essential to confirm its efficacy and safety before clinical application.

## Ethics Statement

All authors listed have made significant contributions to the conception, design, execution, and interpretation of the study. No individuals who contributed to the work have been excluded from authorship. This manuscript is an original work and has not been published or submitted for consideration elsewhere. We confirm that all data presented in this manuscript are accurate and have been collected and analyzed in accordance with accepted standards of scientific research. We are prepared to provide raw data and supporting materials upon request for verification purposes.

## Disclosure

Each author has reviewed and approved the final version of the manuscript and consents to its submission to this journal

## Conflicts of Interest

The authors declare no conflicts of interest.

## Author Contributions

**Pooriya Teimoori:** conceptualization, methodology, investigation, writing – original draft. **Kosar Khatir:** conceptualization, methodology, writing – original draft. **Mohammadreza Heidari:** conceptualization, supervision, methodology, writing – reviewing and editing.

## Funding

No funding was received for this manuscript.

## Supporting information


**Supporting Information** Additional supporting information can be found online in the Supporting Information section. Figure S1: The RMSD profiles (Second analysis). Figure S2: The RMSD profiles (Third analysis). Figure S3: The RMSF profiles (Second analysis). Figure S4: The RMSF profiles (Third analysis). Figure S5: Radius of gyration (Rg) analysis (Second analysis). Figure S6: Radius of gyration (Rg) analysis (Third analysis). Figure S7: Solvent accessible surface area (SASA) analysis (Second analysis). Figure S8: Solvent accessible surface area (SASA) analysis (Third analysis). Figure S9: Hydrogen bond analysis (Second analysis). Figure S10: Hydrogen bond analysis (Third analysis).

## Data Availability

The data that support the findings of this study are available from the corresponding author upon reasonable request.
