# Supplementary material for: Designing a Multi‐Epitope Vaccine Against NOTCH1 and NOTCH4: A Computational Approach for Triple‐Negative Breast Cancer
Source: Biomed Res Int. 2026 Jan 18;2026:9723304. doi: 10.1155/bmri/9723304 (PMC12812874; doi:10.1155/bmri/9723304)
Supplement: Supplementary file 1 — Supporting Information Additional supporting information can be found online in the Supporting Information section. Figure S1: The RMSD profiles (Second analysis). Figure S2: The RMSD profiles (Third analysis). Figure S3: The RMSF profiles (Second analysis). Figure S4: The RMSF profiles (Third analysis). Figure S5: Radius of gyration (Rg) analysis (Second analysis). Figure S6: Radius of gyration (Rg) analysis (Third analysis). Figure S7: Solvent accessible surface area (SASA) analysis (Second analysis). Figure S8: Solvent accessible surface area (SASA) analysis (Third analysis). Figure S9: Hydrogen bond analysis (Second analysis). Figure S10: Hydrogen bond analysis (Third analysis). [file BMRI-2026-9723304-s001.docx]

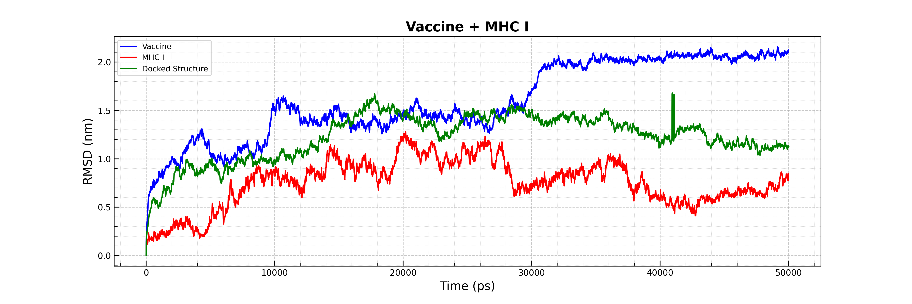

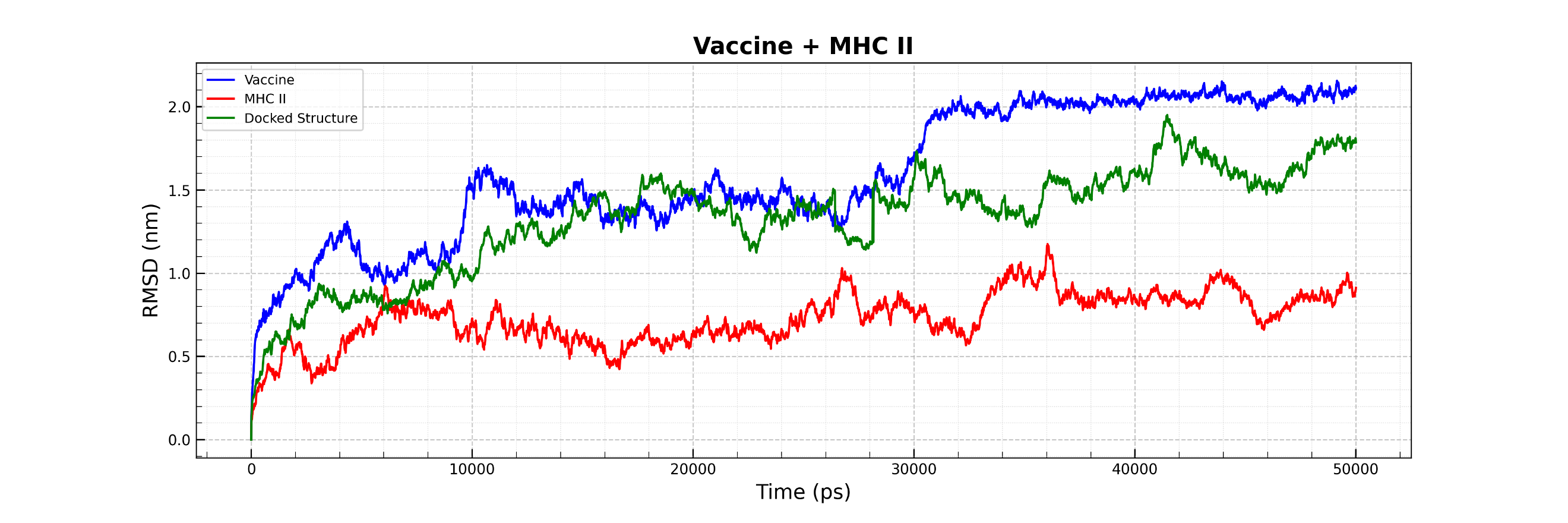

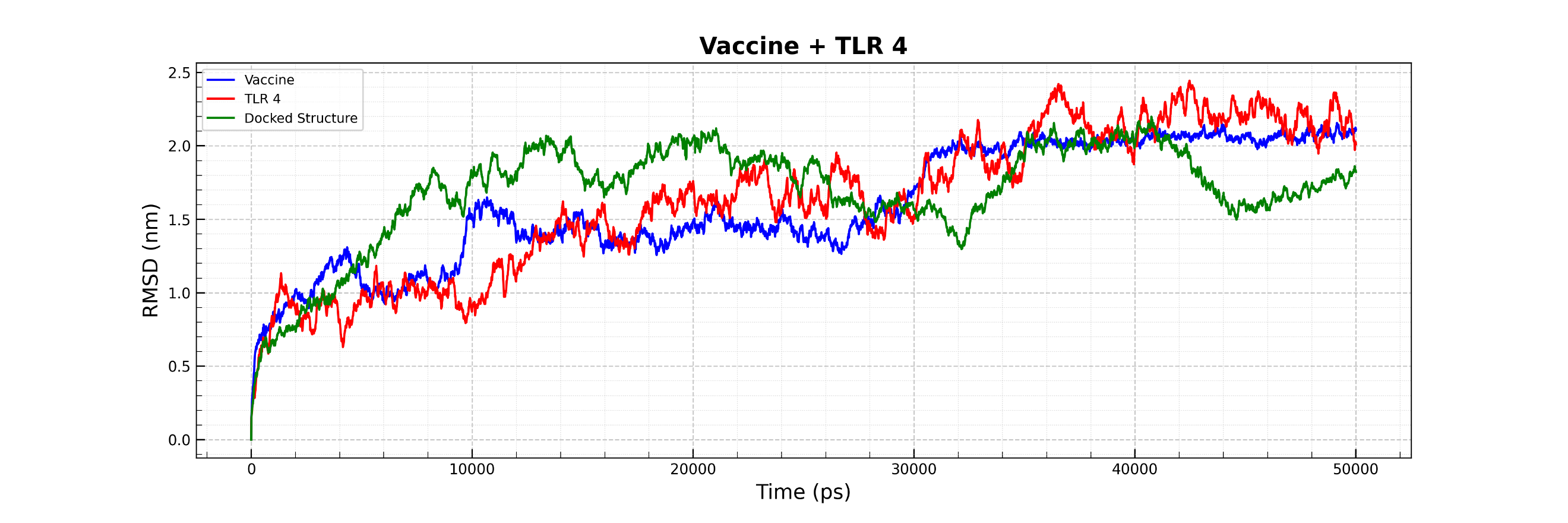


Figure S1. The RMSD profiles for the docked complexes (Green) alongside the individual Multi-Epitope Vaccine (Blue) and immune receptors MHC I, MHC II, and TLR4 (Red) from the MD simulation analysis. (Second Analysis)


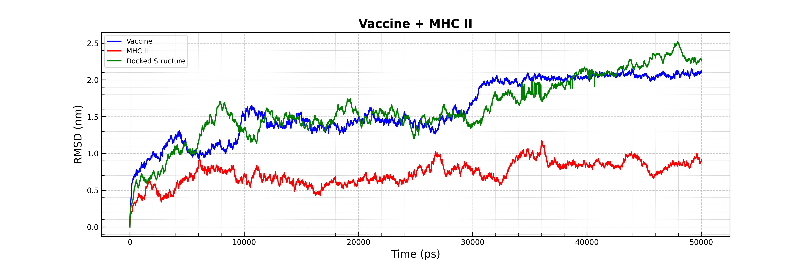

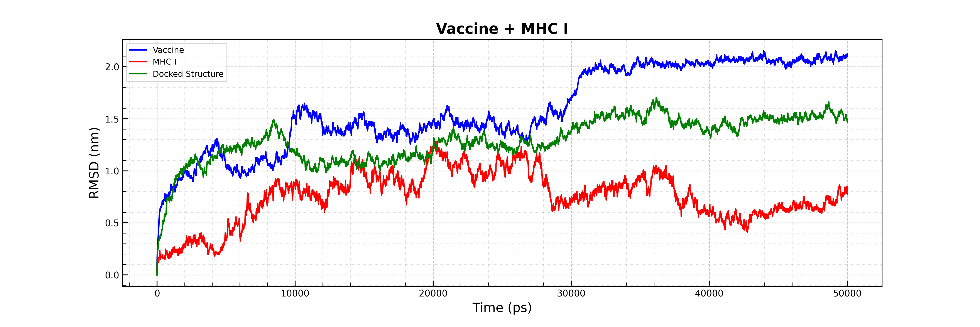

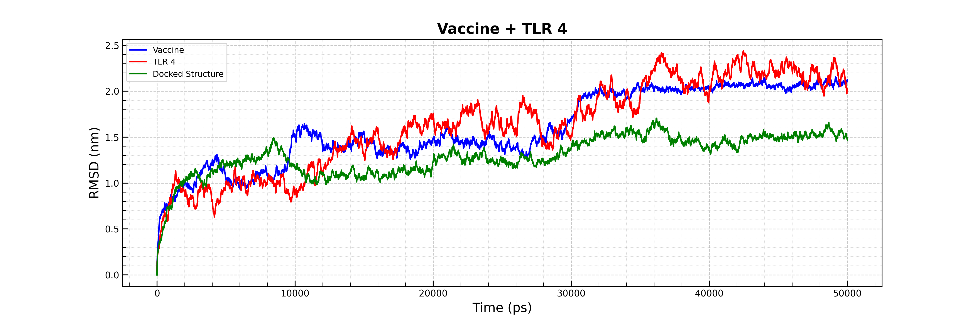


Figure S2. The RMSD profiles for the docked complexes (Green) alongside the individual Multi-Epitope Vaccine (Blue) and immune receptors MHC I, MHC II, and TLR4 (Red) from the MD simulation analysis. (Third Analysis)

Figure S3. The RMSF profiles for the docked complexes (Green) alongside the individual Multi-Epitope Vaccine (Blue) and immune receptors MHC I, MHC II, and TLR4 (Red) from the MD simulation analysis. (Second Analysis)


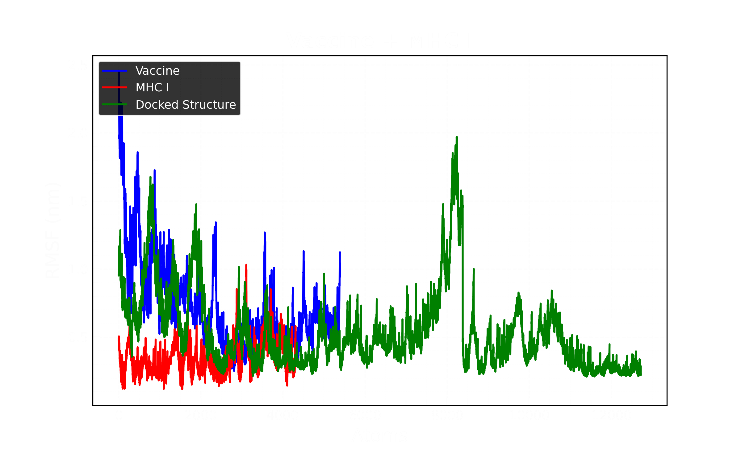

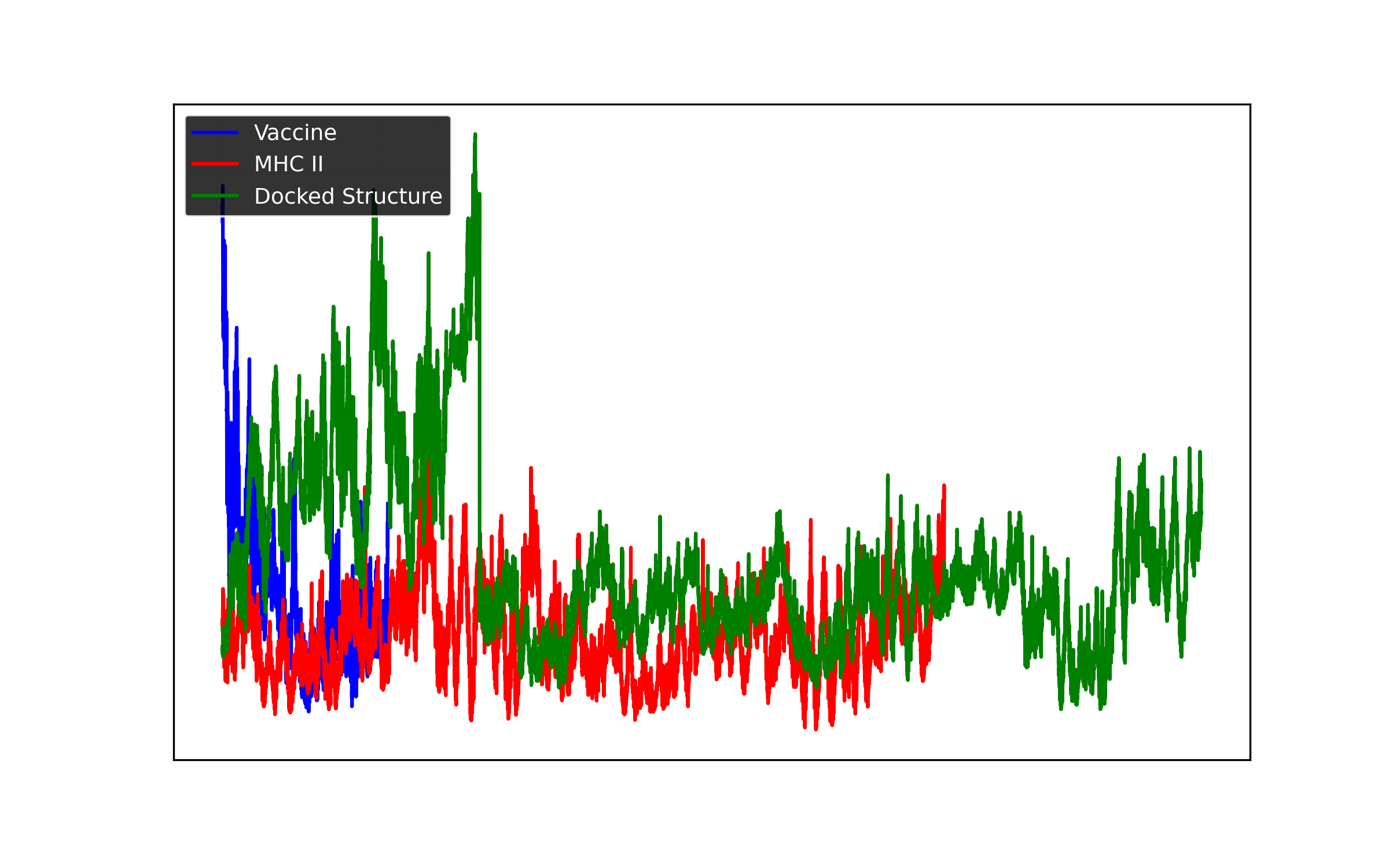

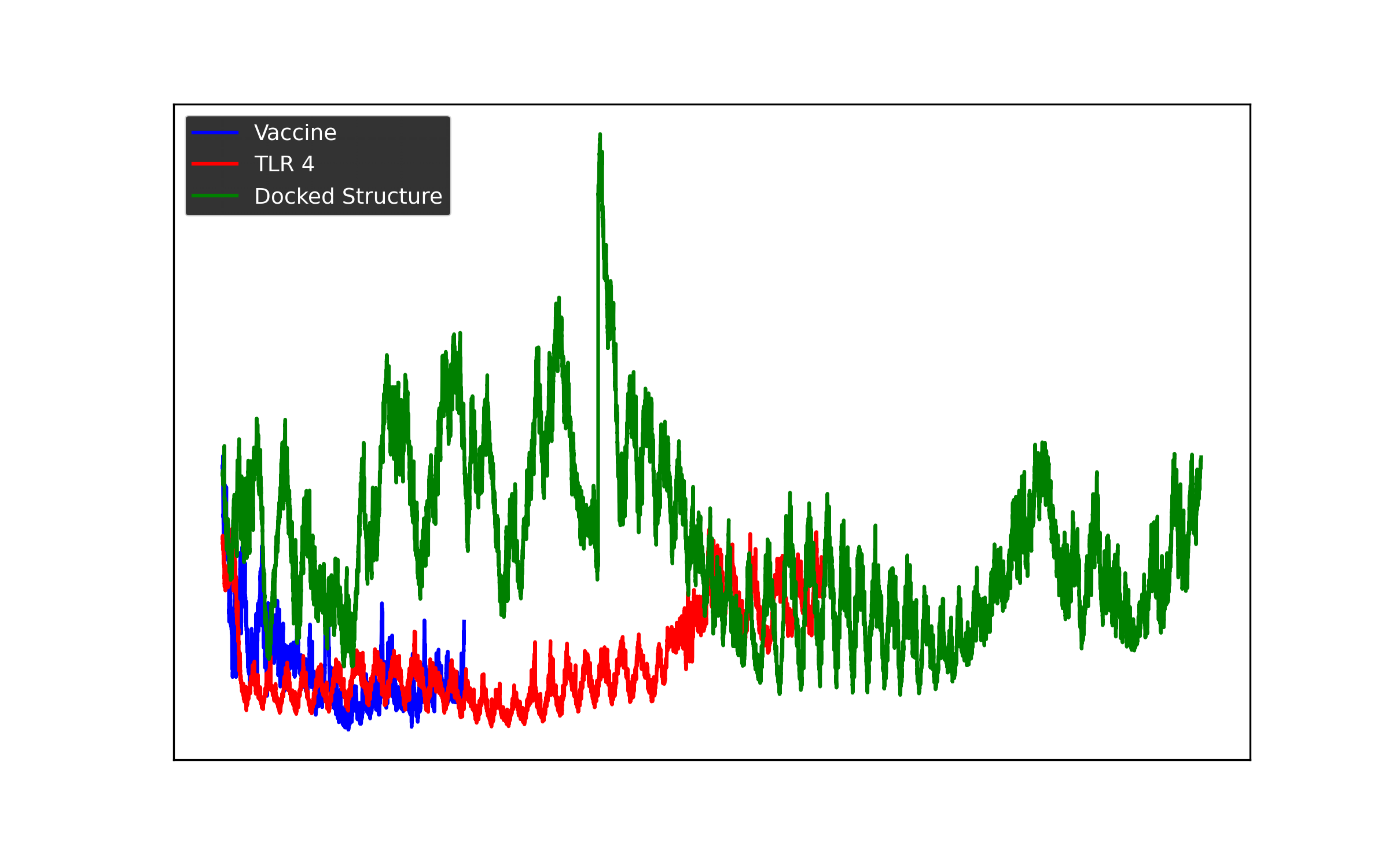

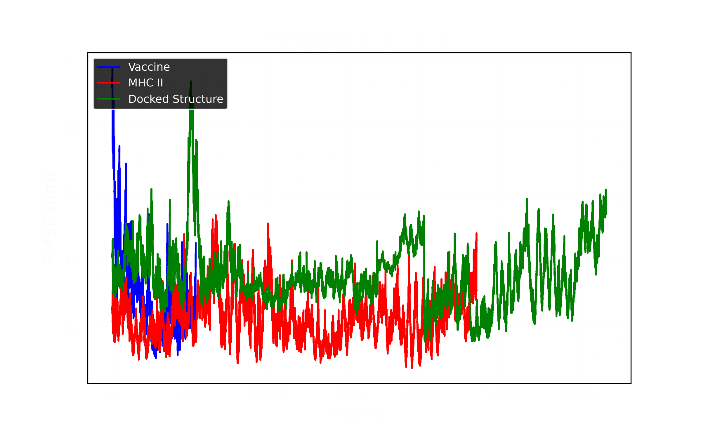

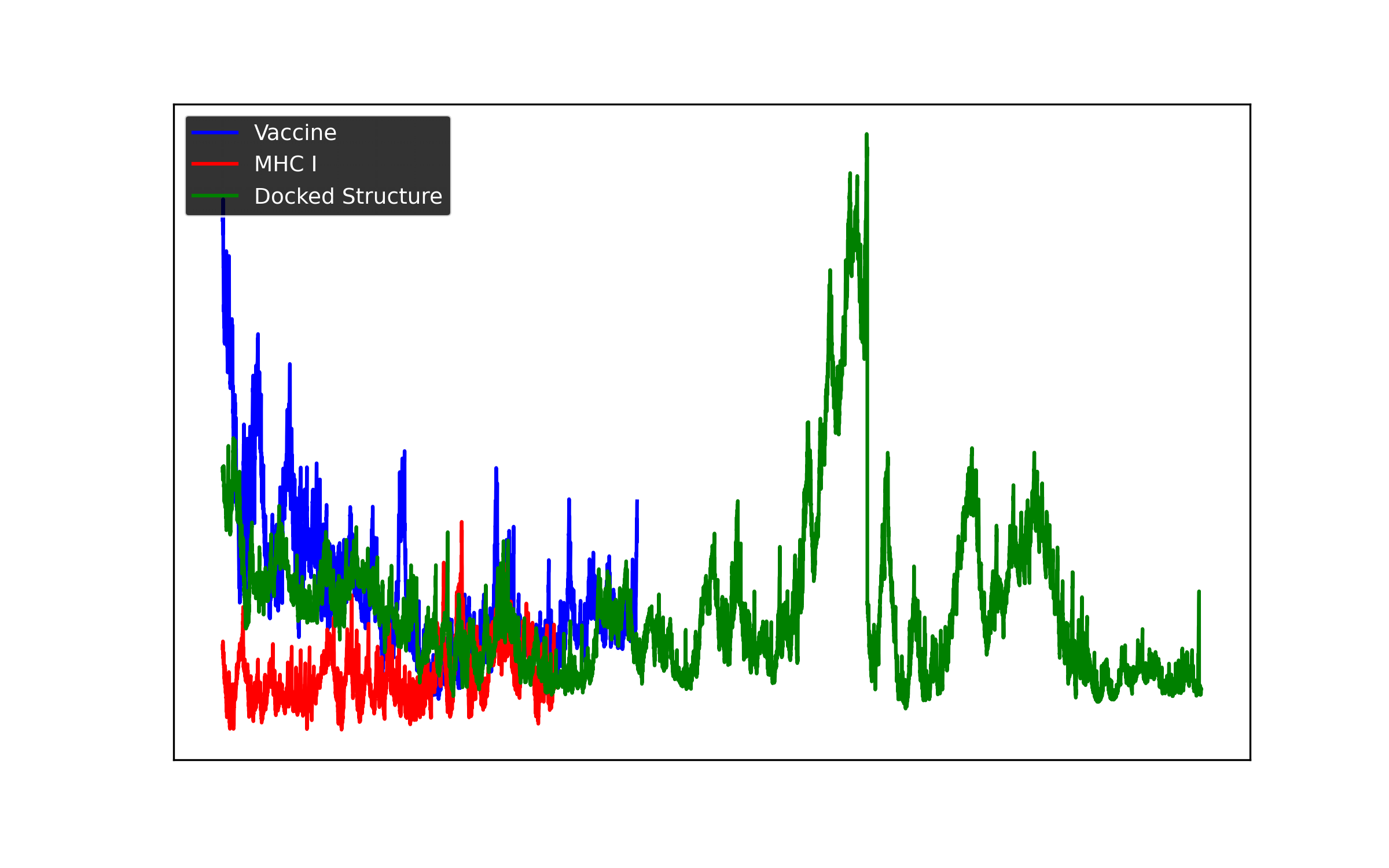

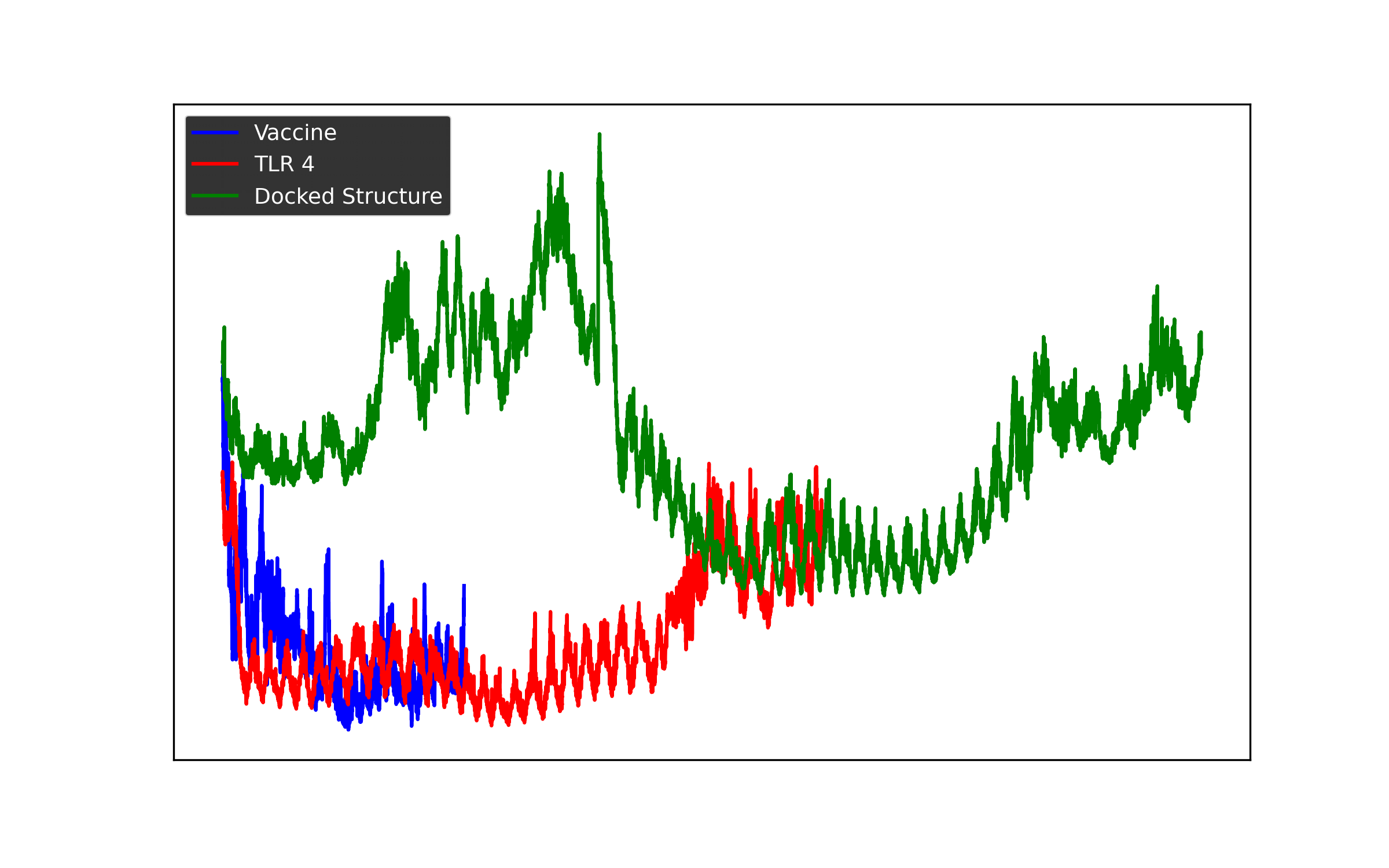


Figure S4. The RMSF profiles for the docked complexes (Green) alongside the individual Multi-Epitope Vaccine (Blue) and immune receptors MHC I, MHC II, and TLR4 (Red) from the MD simulation analysis. (Third Analysis)

Figure S5. Radius of Gyration (Rg) analysis of the vaccine in complex with MHC I, MHC II, and TLR4 over a 50,000 ps simulation. (Second Analysis)


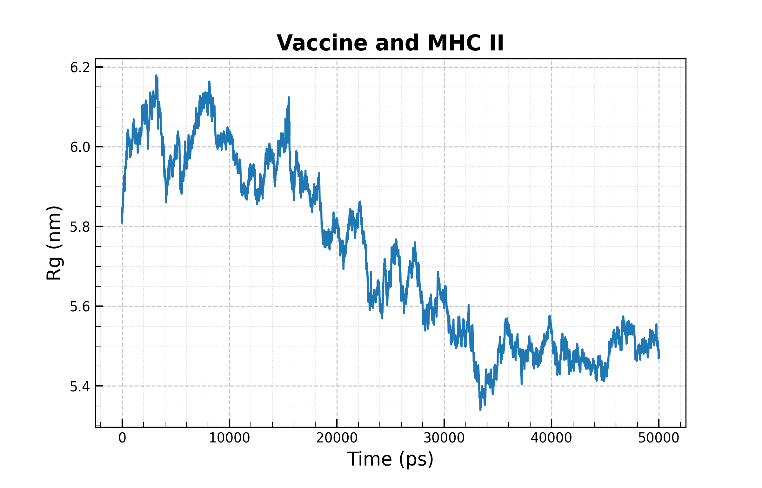

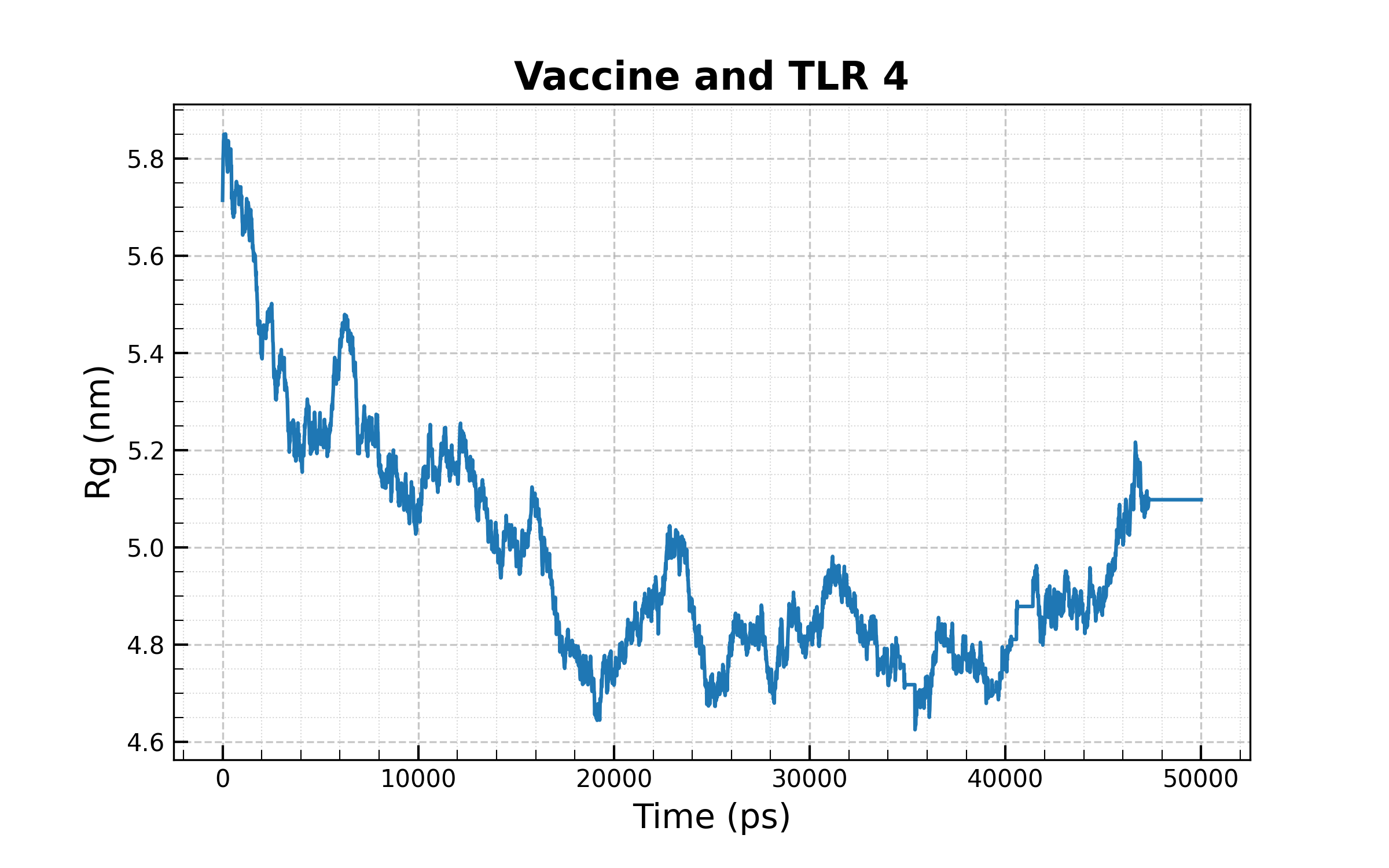

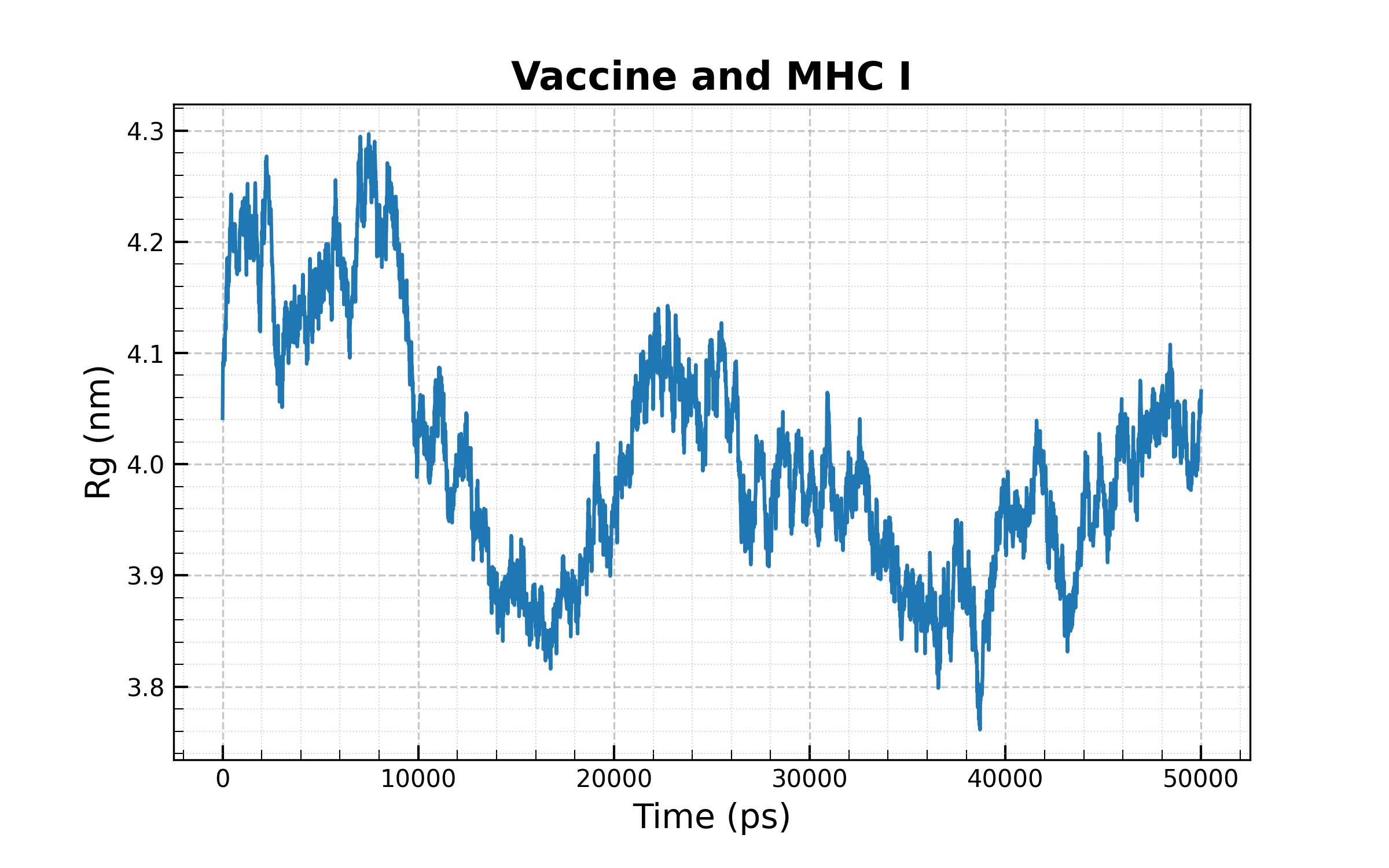

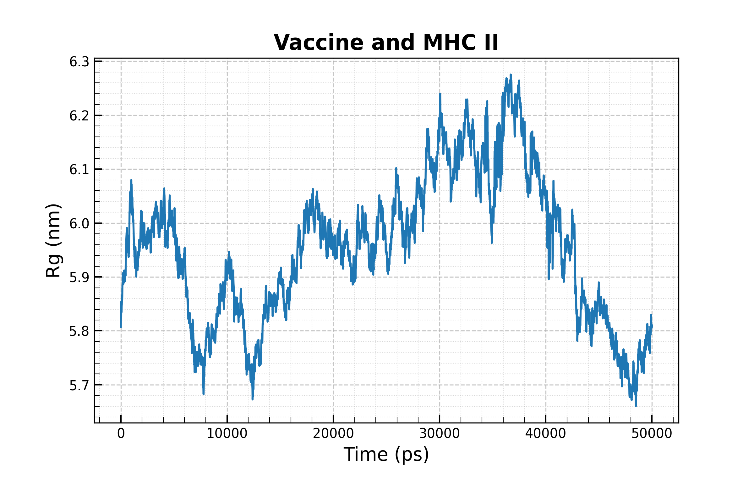

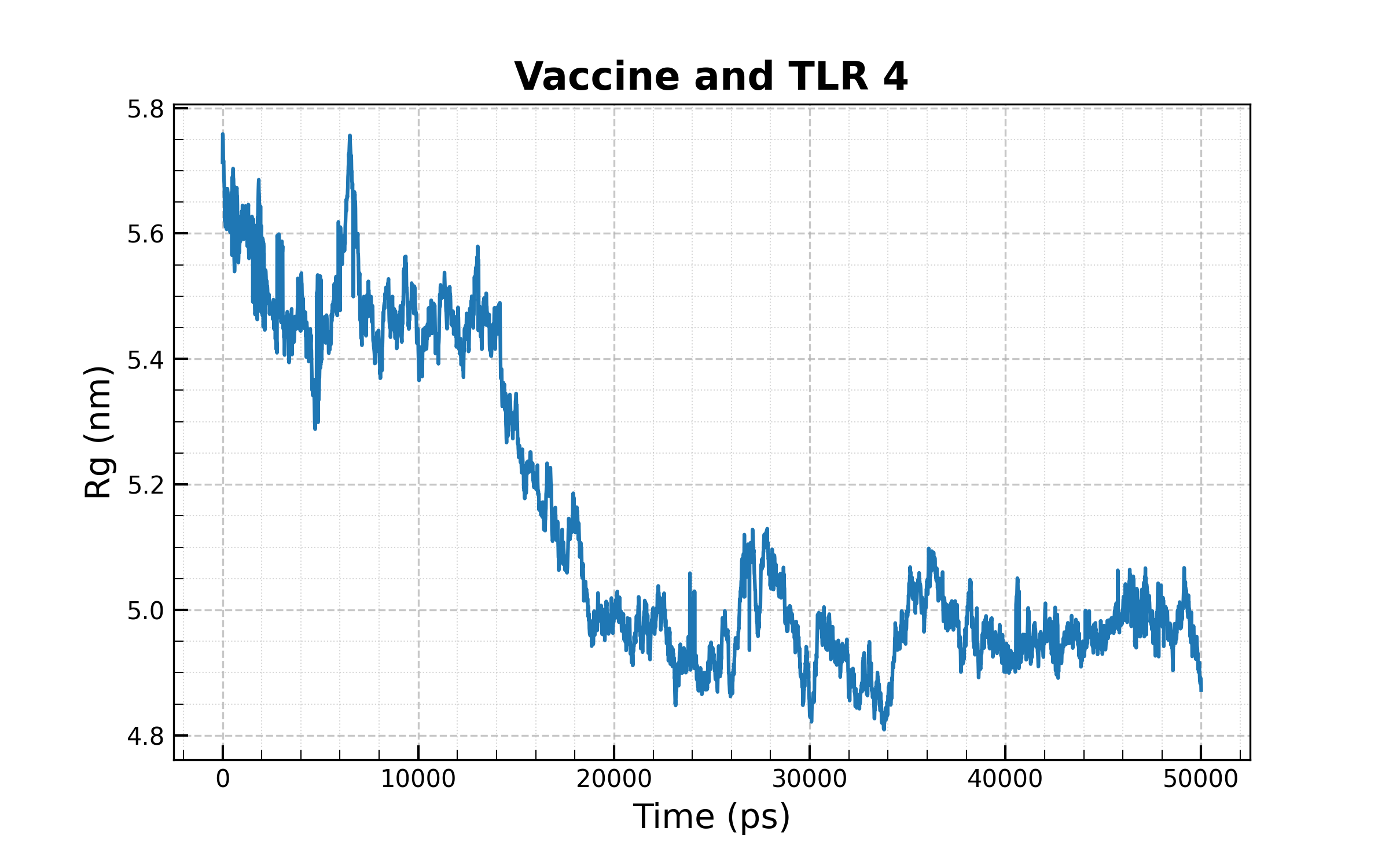

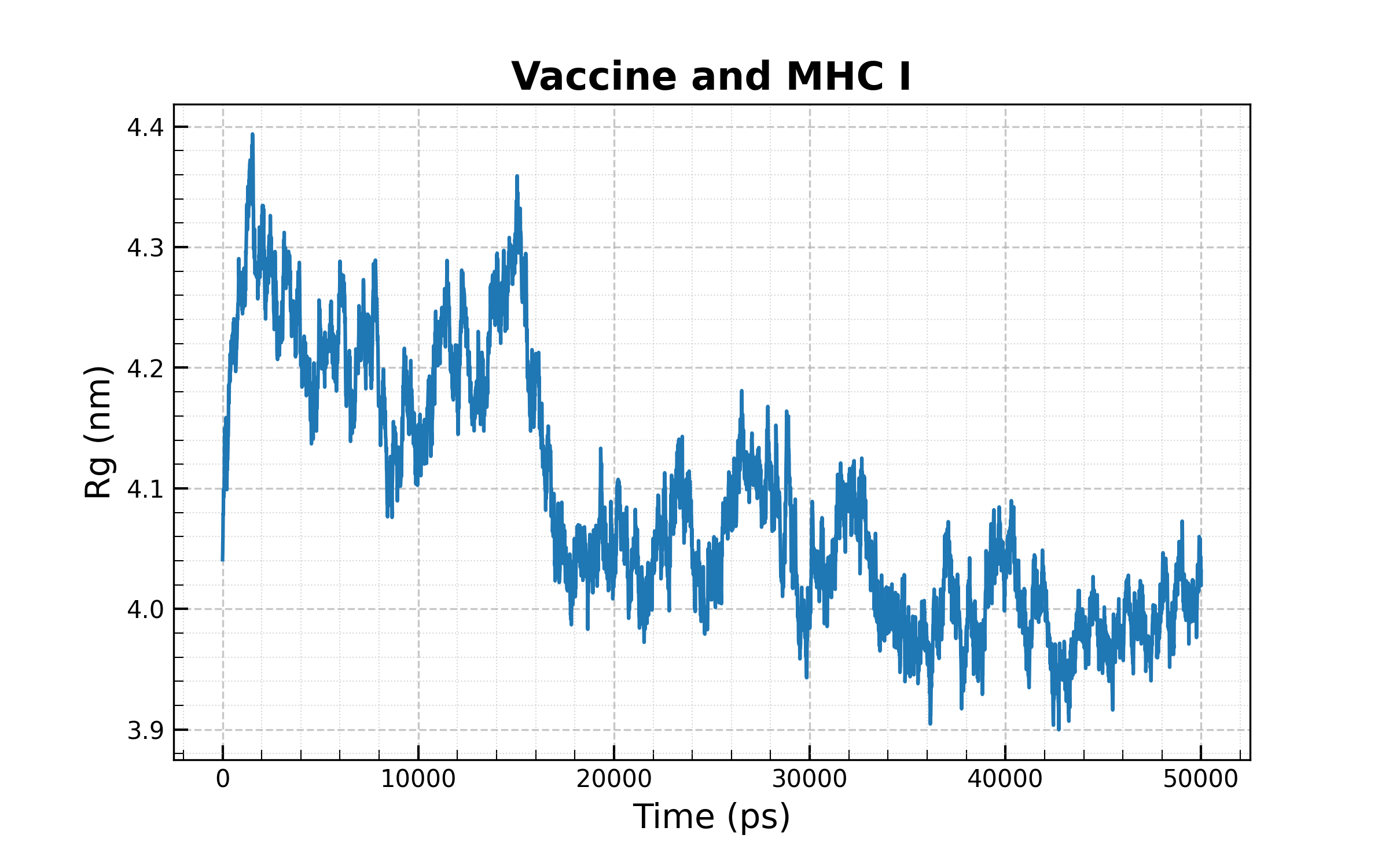


Figure S6. Radius of Gyration (Rg) analysis of the vaccine in complex with MHC I, MHC II, and TLR4 over a 50,000 ps simulation. (Third Analysis)

Figure S7. Solvent Accessible Surface Area (SASA) analysis of the vaccine complexed with MHC I, MHC II, and TLR4. (Second Analysis)


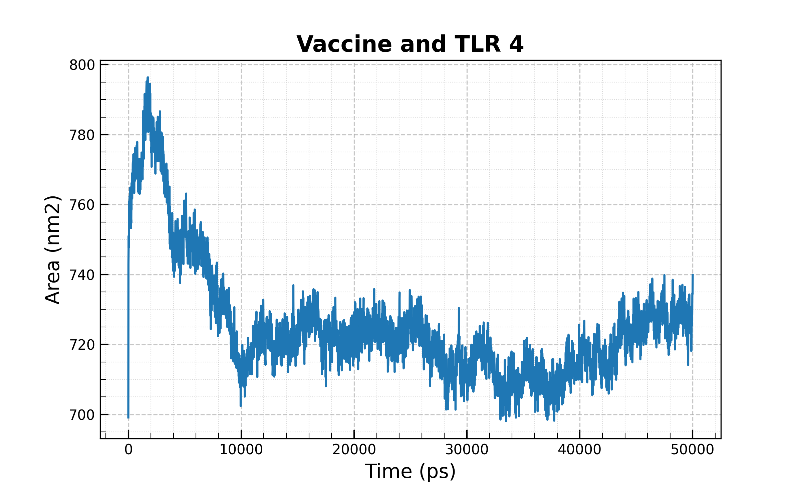

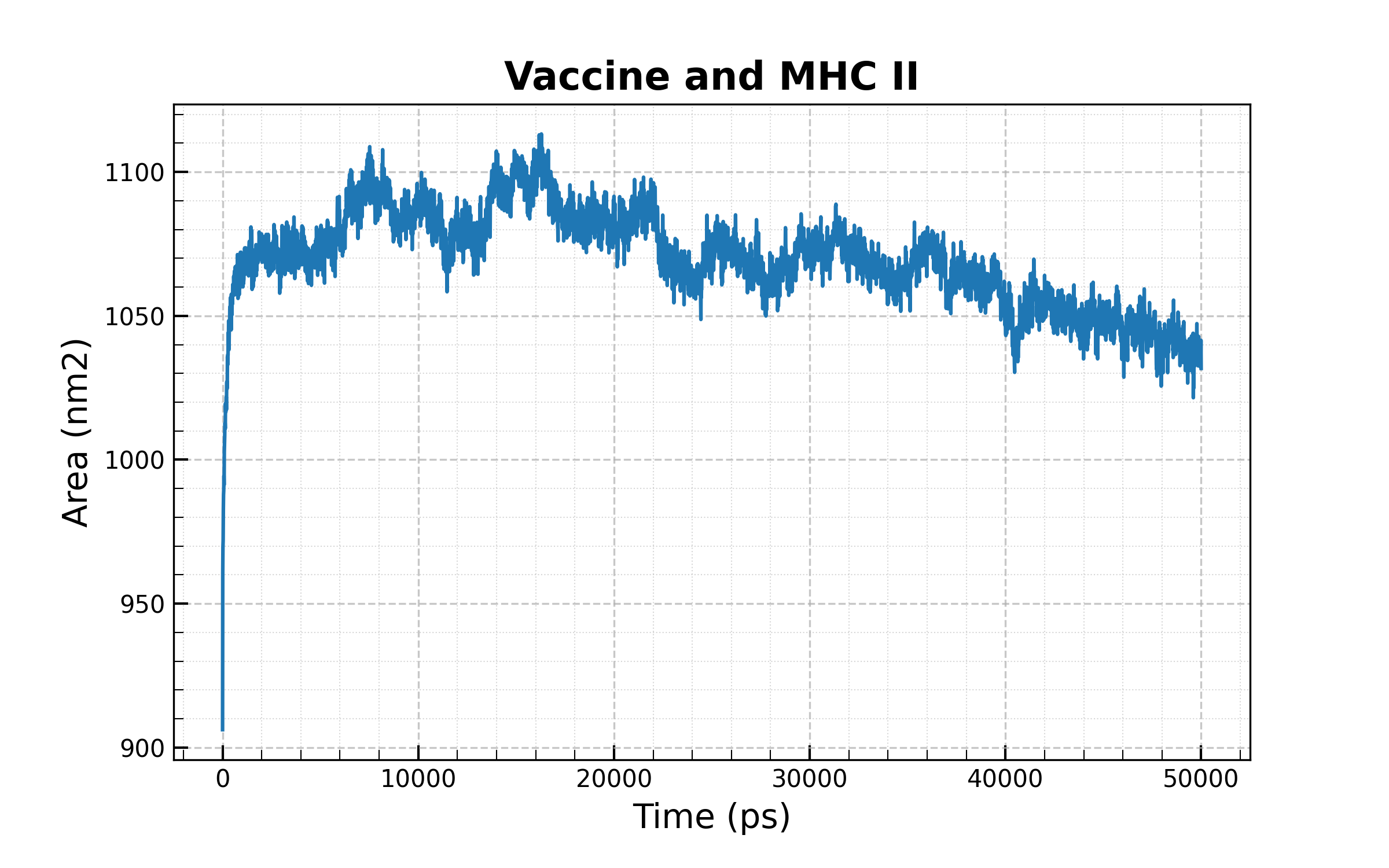

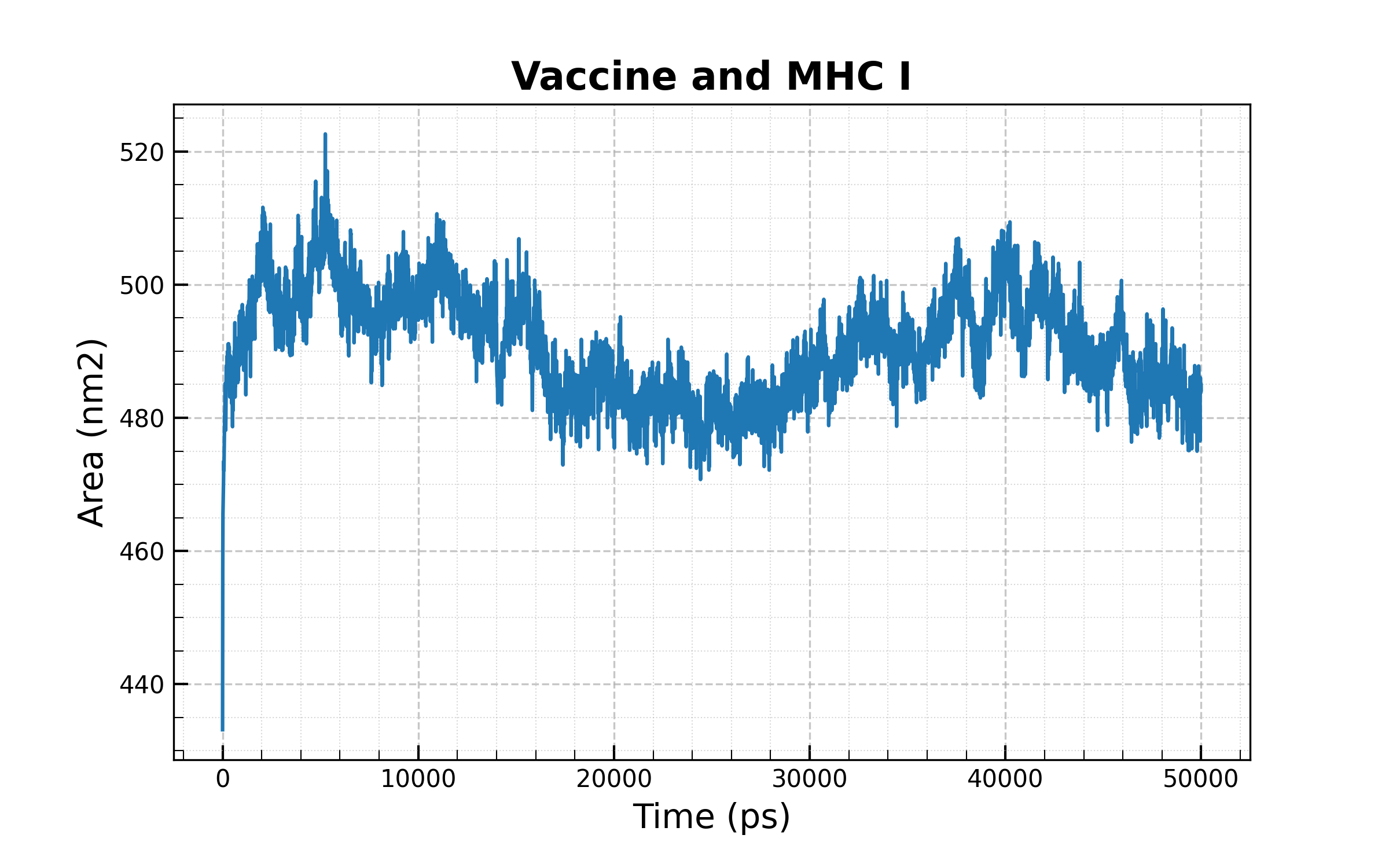

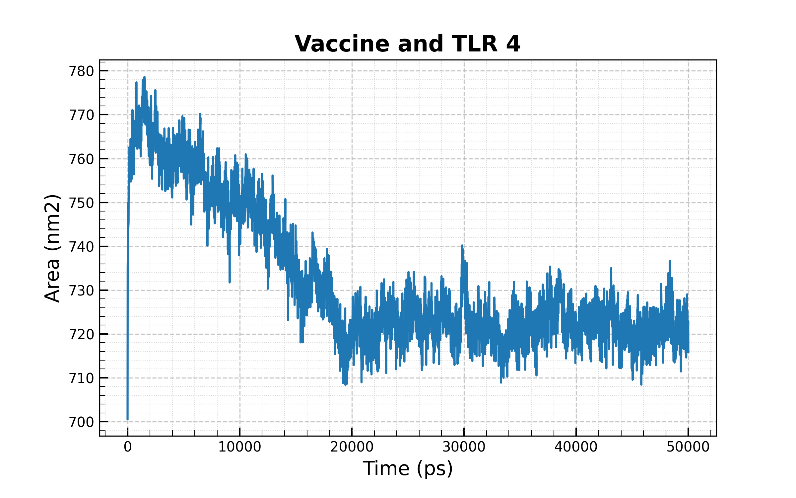

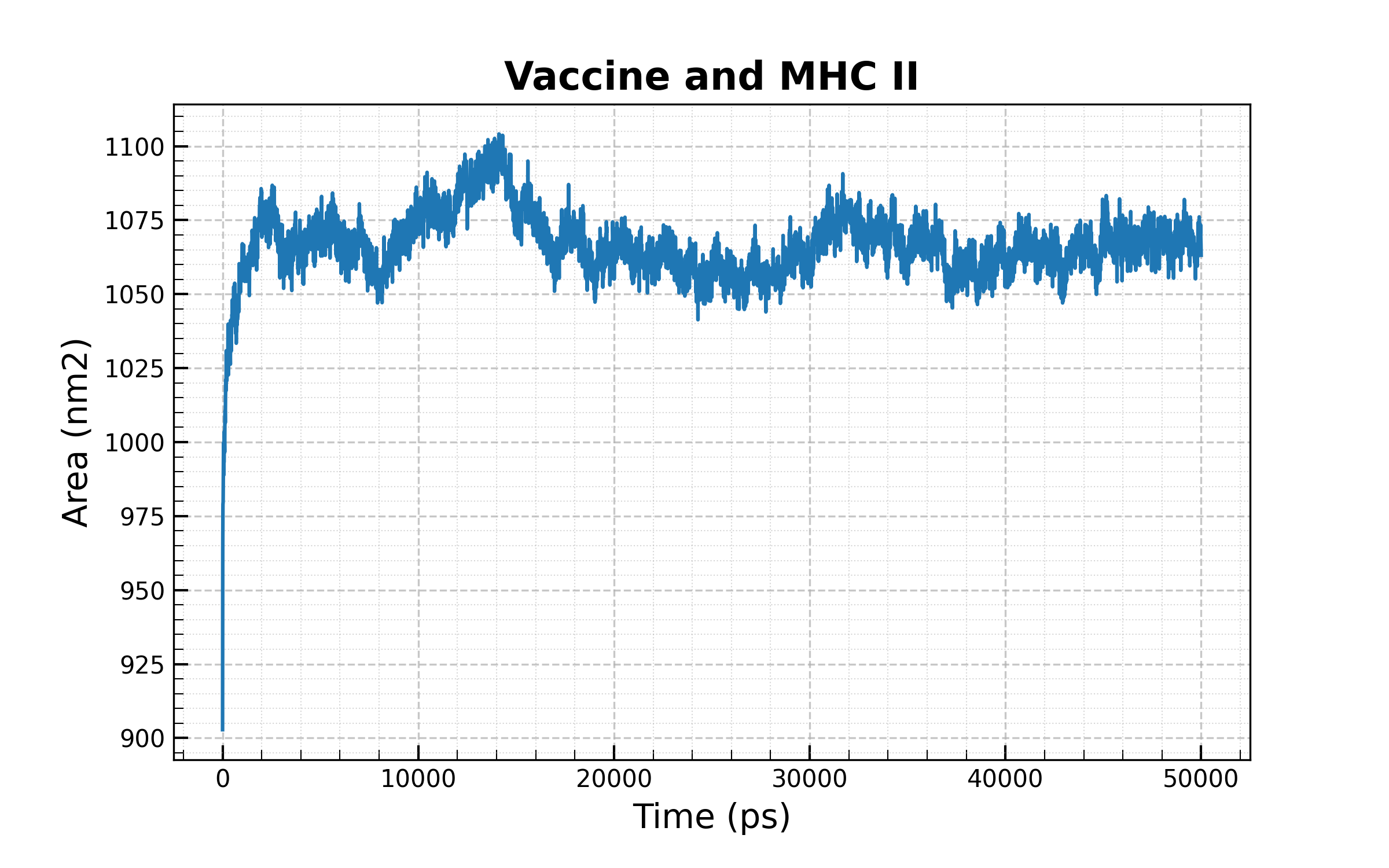

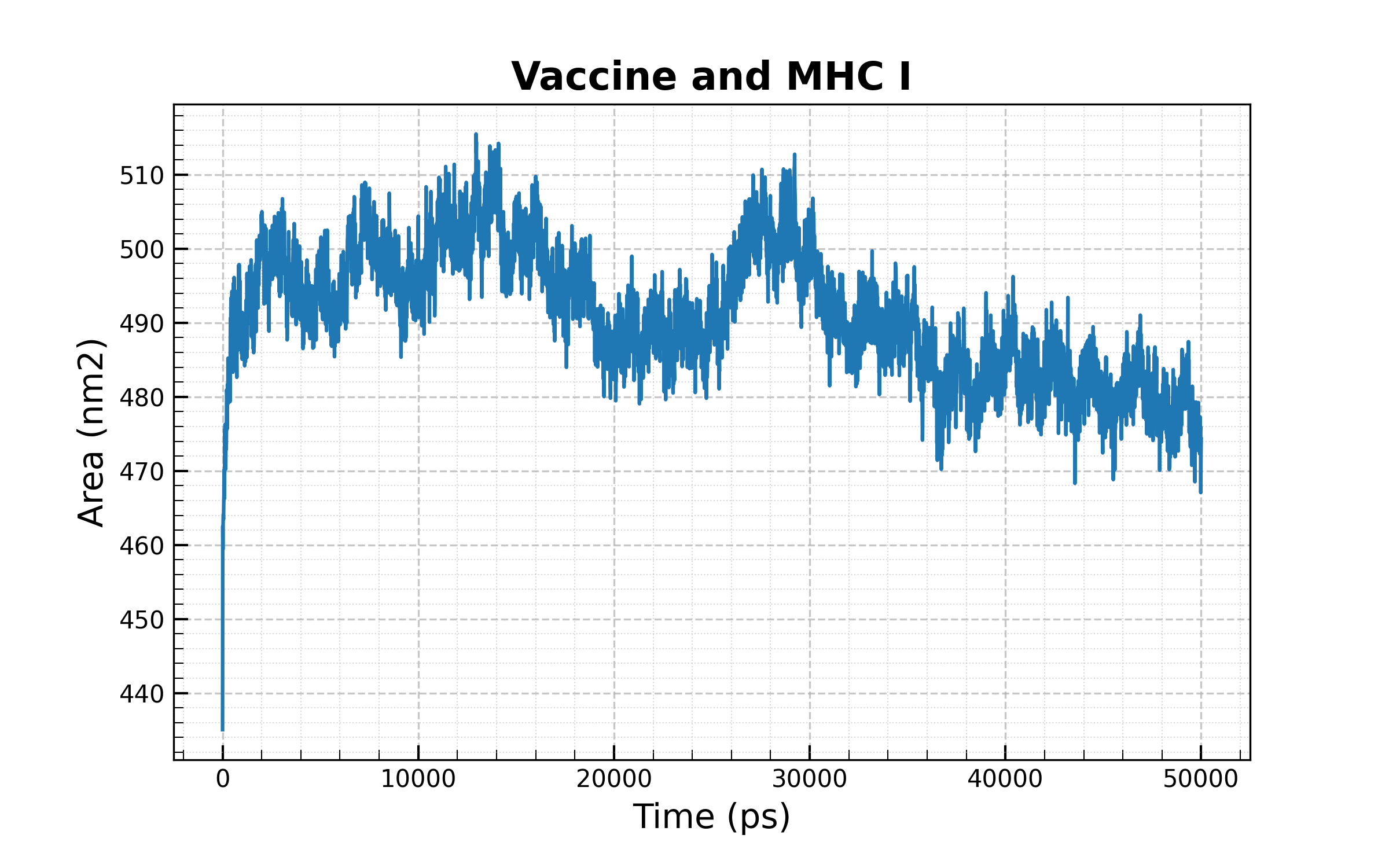


Figure S8. Solvent Accessible Surface Area (SASA) analysis of the vaccine complexed with MHC I, MHC II, and TLR4. (Third Analysis)

Figure S9. Hydrogen bond analysis of vaccine interactions with MHC I, MHC II, and TLR4. (Second Analysis)


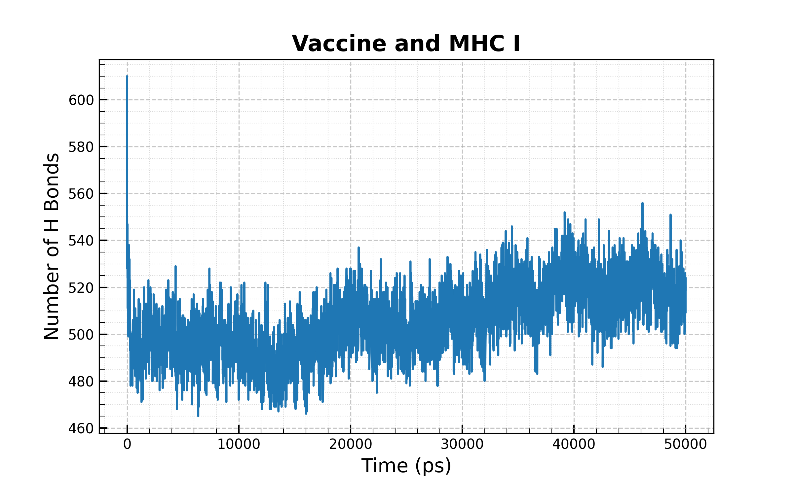

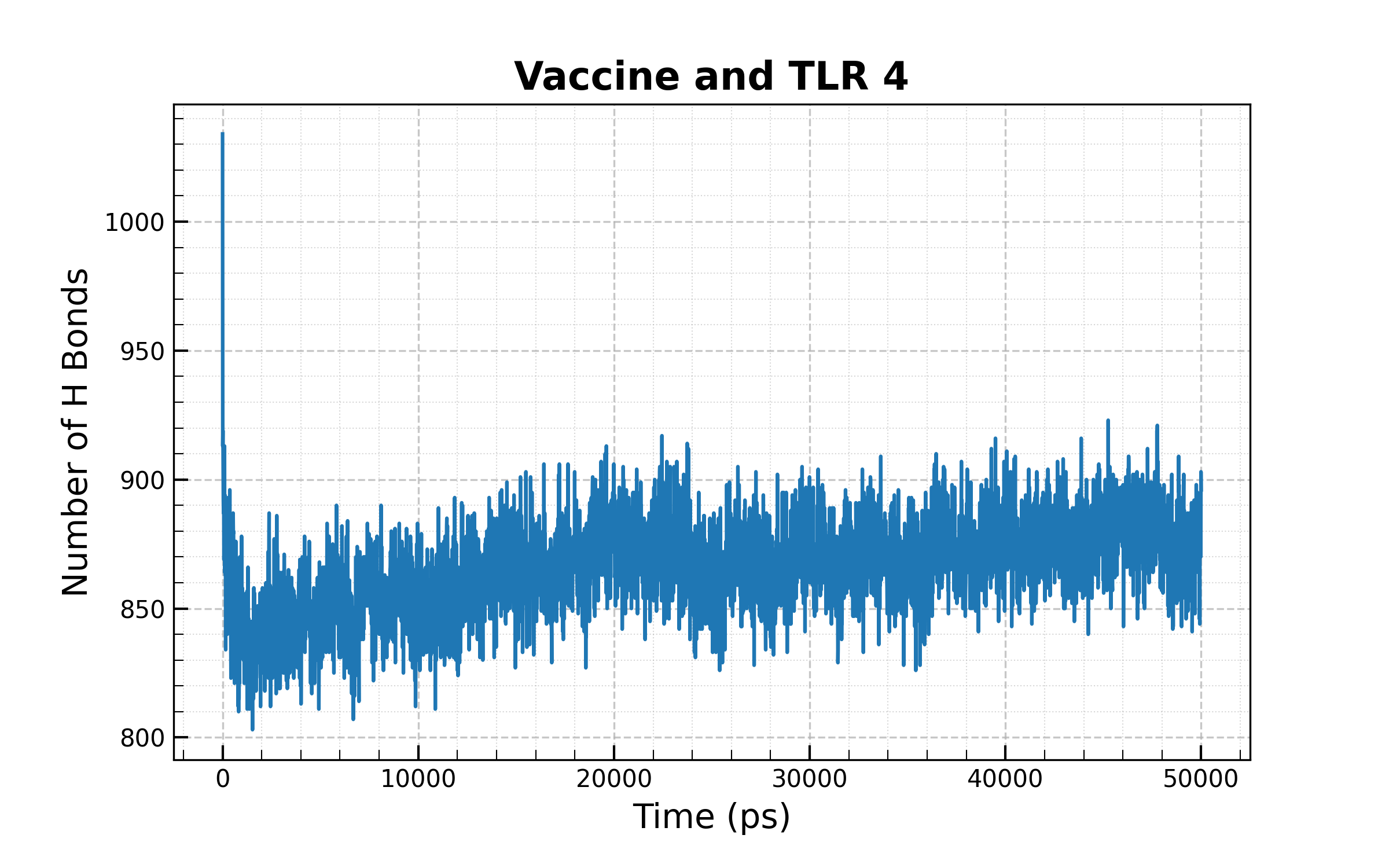

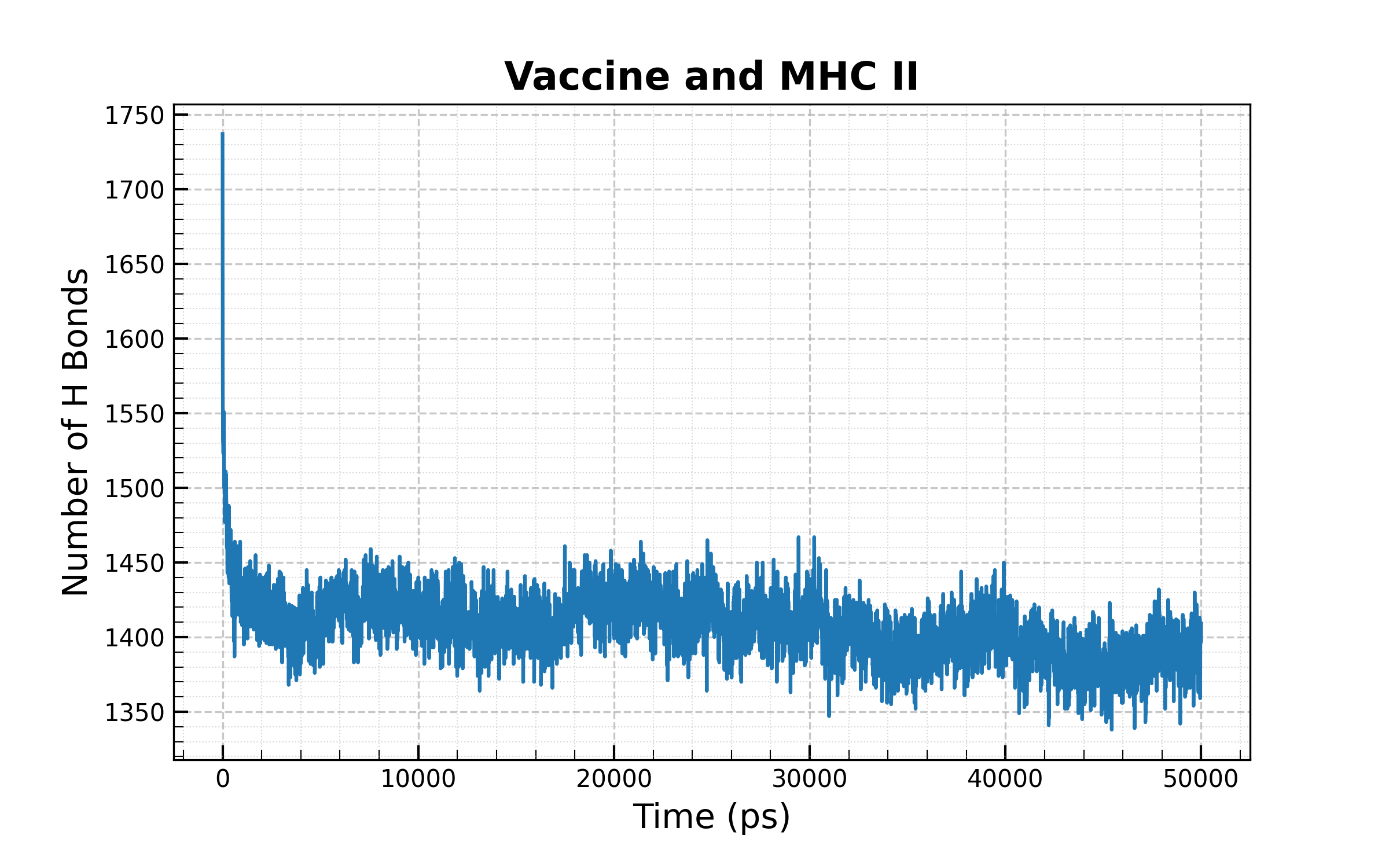

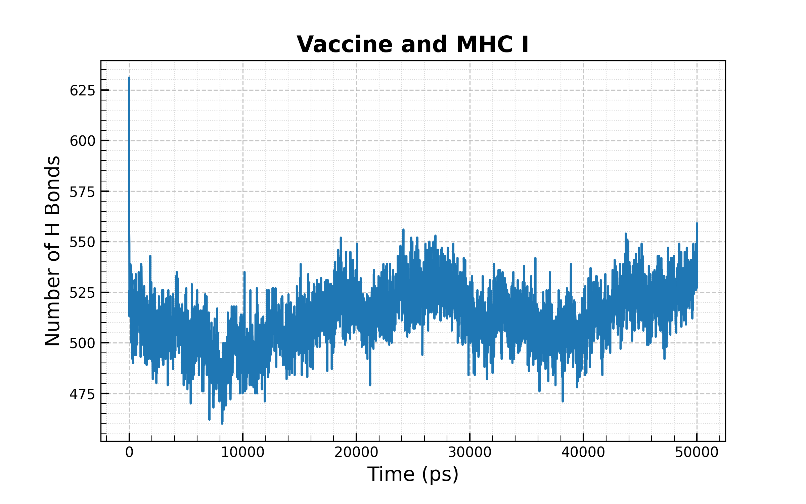

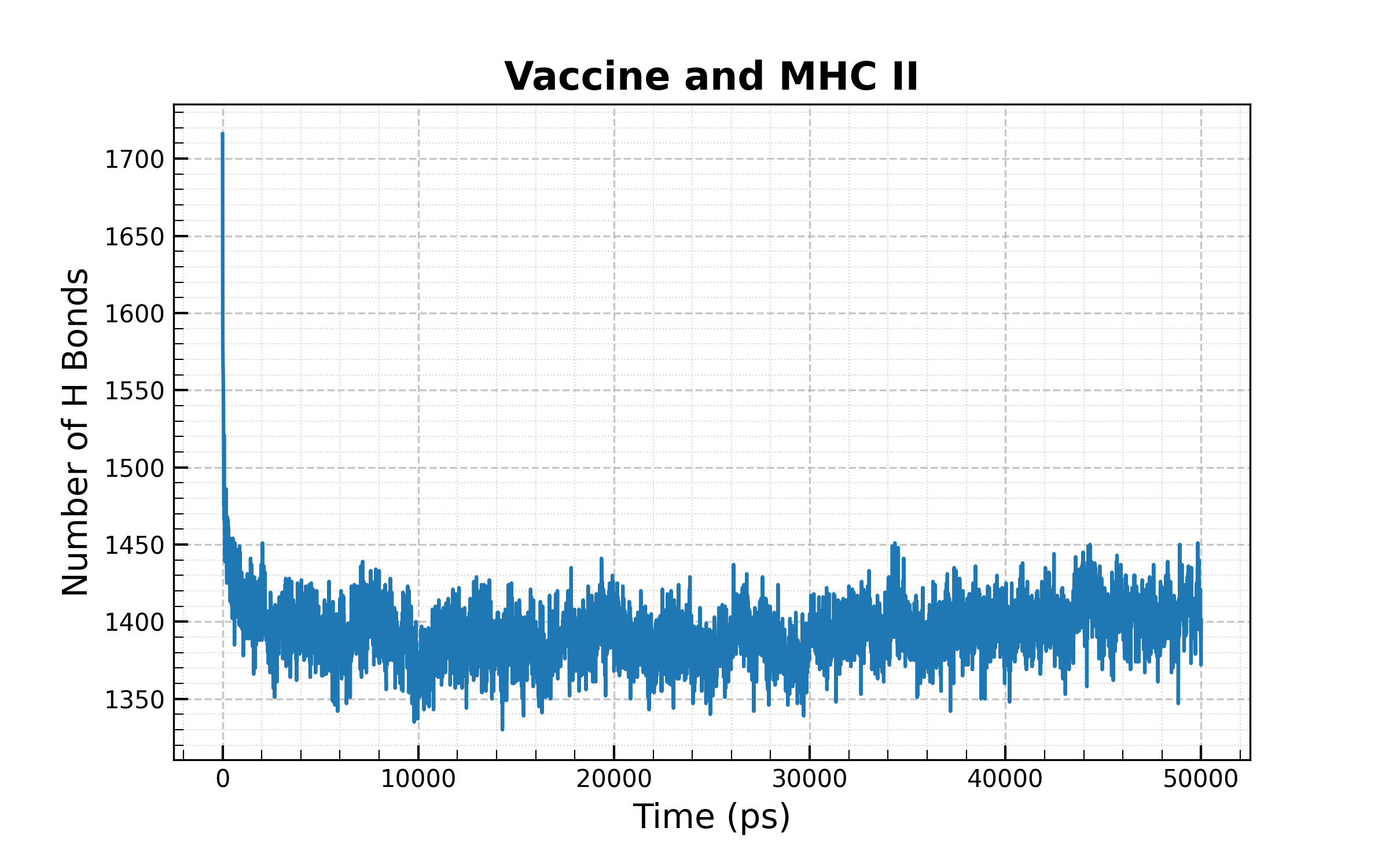

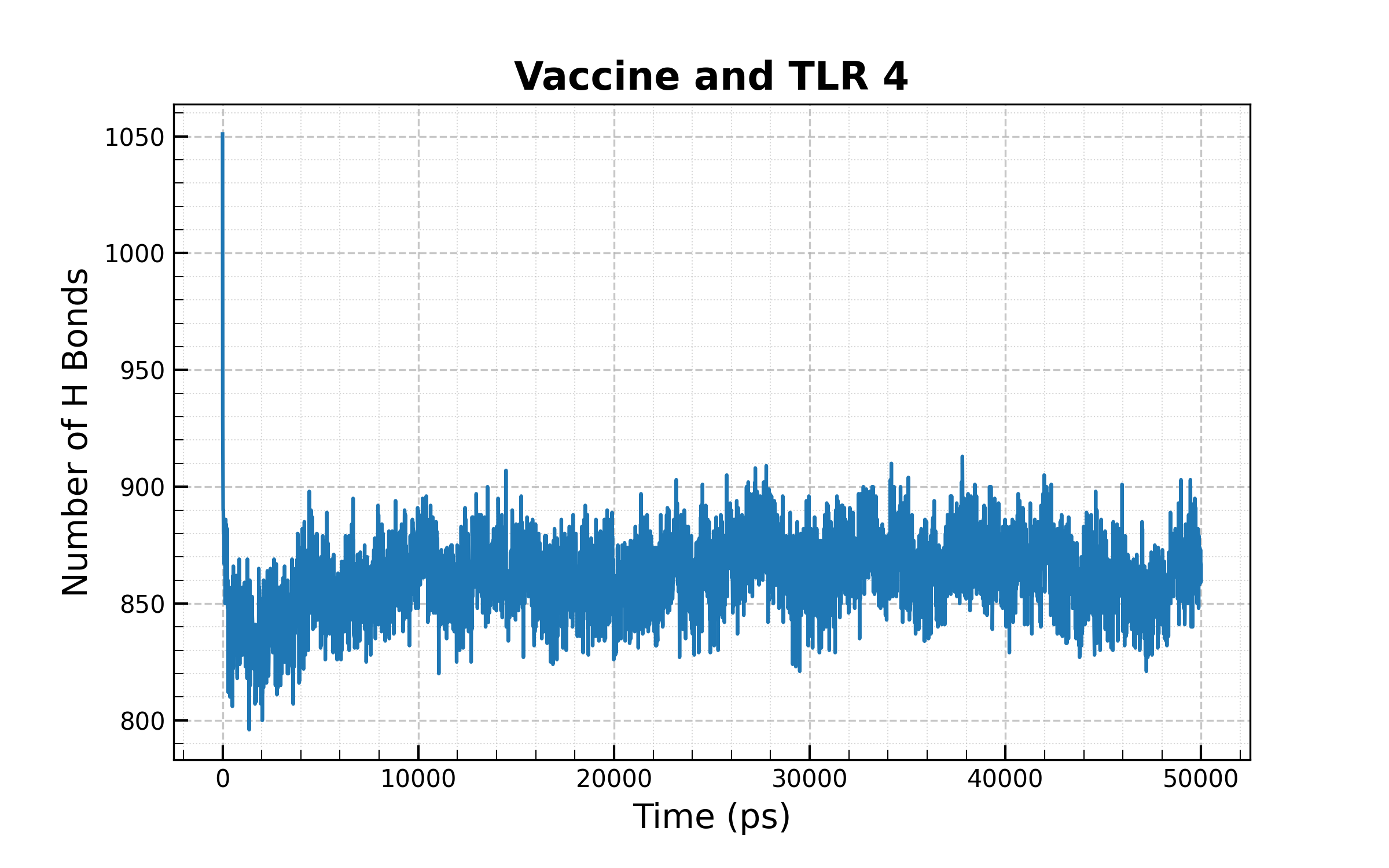


Figure S10. Hydrogen bond analysis of vaccine interactions with MHC I, MHC II, and TLR4. (Third Analysis)
